# Operative and peri‐operative considerations in the management of brain metastasis

**DOI:** 10.1002/cam4.2577

**Published:** 2019-09-30

**Authors:** Eric W. Sankey, Vadim Tsvankin, Matthew M. Grabowski, Gautam Nayar, Kristen A. Batich, Aida Risman, Cosette D. Champion, April K. S. Salama, C. Rory Goodwin, Peter E. Fecci

**Affiliations:** ^1^ Department of Neurosurgery Duke University Medical Center Durham NC USA; ^2^ Department of Neurosurgery Cleveland Clinic Cleveland OH USA; ^3^ Department of Neurosurgery University of Pittsburgh Medical Center Pittsburgh PA USA; ^4^ Department of Medicine Duke University Medical Center Durham NC USA; ^5^ School of Medicine Medical College of Georgia Augusta GA USA; ^6^ Division of Medical Oncology Duke University Medical Center Durham NC USA

**Keywords:** cancer management, metastasis, radiation therapy, surgical therapy

## Abstract

The number of patients who develop metastatic brain lesions is increasing as the diagnosis and treatment of systemic cancers continues to improve, resulting in longer patient survival. The role of surgery in the management of brain metastasis (BM), particularly multiple and recurrent metastases, remains controversial and continues to evolve. However, with appropriate patient selection, outcomes after surgery are typically favorable. In addition, surgery is the only means to obtain a tissue diagnosis and is the only effective treatment modality to quickly relieve neurological complications or life‐threatening symptoms related to significant mass effect, CSF obstruction, and peritumoral edema. As such, a thorough understanding of the role of surgery in patients with metastatic brain lesions, as well as the factors associated with surgical outcomes, is essential for the effective management of this unique and growing patient population.

## Graphical abstract

The number of patients who develop brain metastases is increasing as improved therapies for systemic cancer prolong survival. Surgery plays an important role in the management of brain metastasis. This review outlines the current data and recommendations for approaching the operative management of brain metastasis.

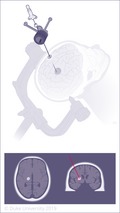

## 1 INTRODUCTION

Of the roughly 1.44 million patients who will be diagnosed with cancer in the United States this year, between 180 000 and 216 000—up to 15%—will be diagnosed with brain metastases (BM).^1^, ^2^ BM have an incidence and mortality greater than any individual malignancy, and have been found in nearly half of patients with systemic malignancies at the time of autopsy.^1^, ^3^ This incidence is approximately 20‐fold higher than glioblastoma, the most common primary brain cancer, and nearly 3‐fold higher than the incidence of all primary brain tumors combined.^4^, ^5^ Moreover, the prevalence of BM is expected to increase as improvements in population heath, cancer screening and systemic treatments result in earlier diagnosis and longer patient survival.^6^, ^7^

**Figure 1:**
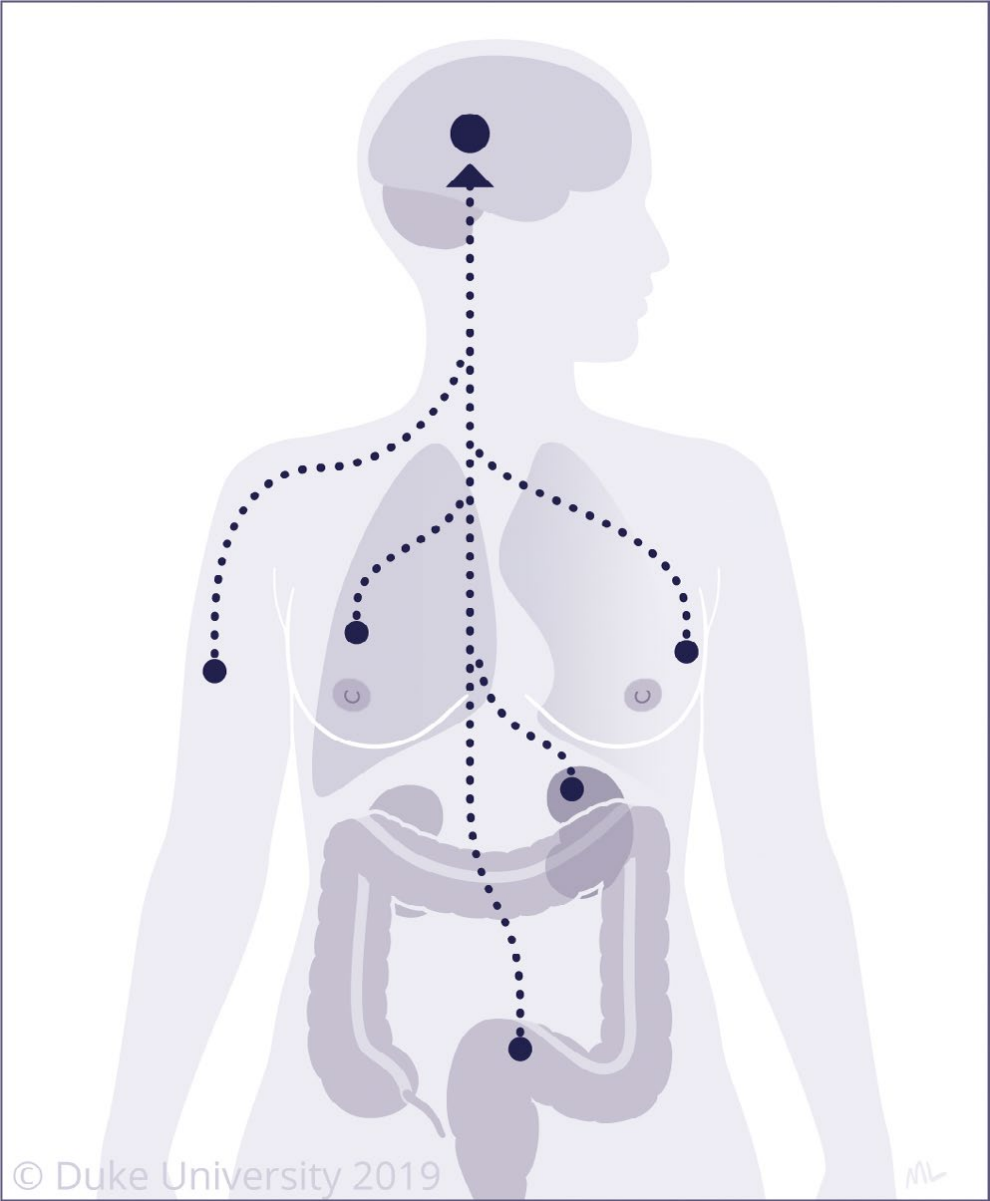
The most common primary cancers metastasizing to the brain include lung, breast, melanoma, colorectal, and renal cell cancer

**Figure 2:**
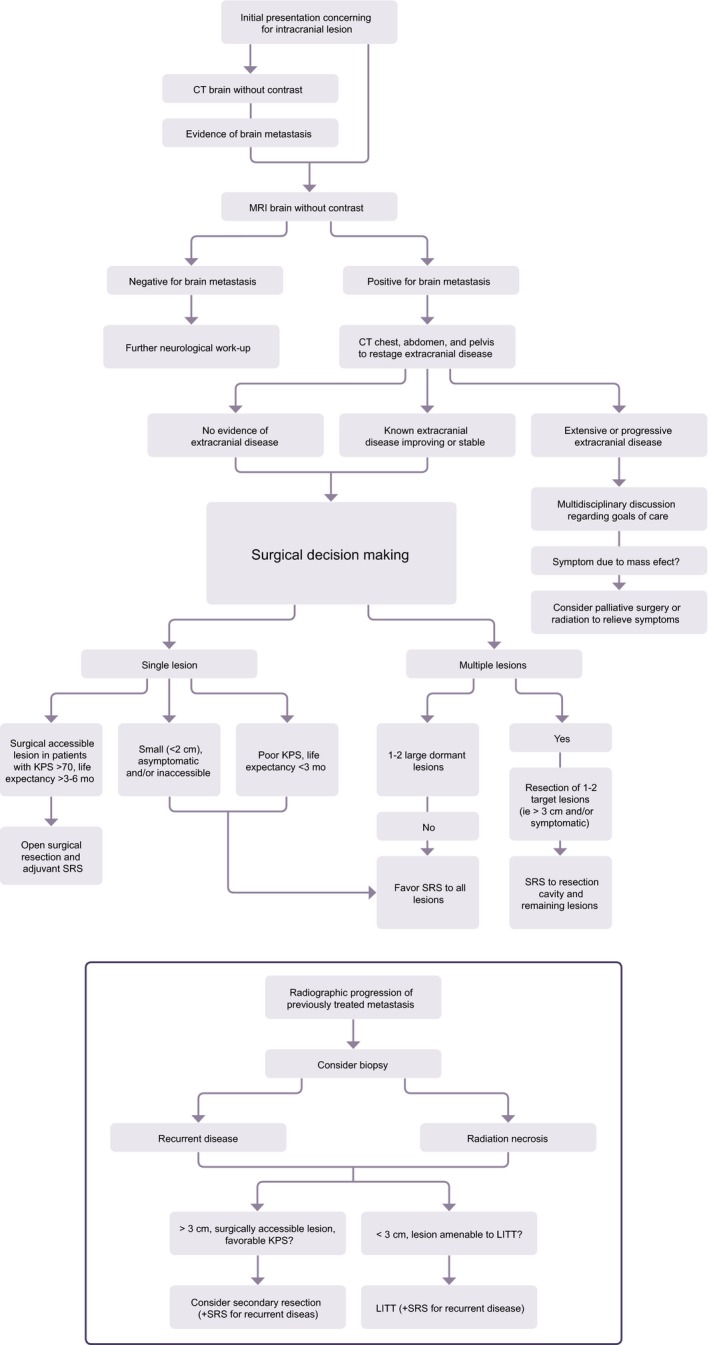
Proposed evaluation and treatment algorithm for management of newly diagnosed brain metastases as well as recurrent lesions

The risk of BM varies considerably between primary cancer types. To date, the most common primary cancers with a proclivity for brain metastasis are lung (50%‐60%), breast (15%‐20%), and melanoma (5%‐10%), followed by kidney (i.e. renal cell carcinoma), colon, pancreas, and other urologic/gynecologic cancers (Figure 1).^8^, ^9^, ^10^ Approximately 80% of BM are located in the cerebral hemispheres, 15% in the cerebellum, and 5% in the brainstem, with 10 to 15% being located in deep^1^ or eloquent^2^ areas of the brain.^5^ Patients with well‐controlled intracranial metastases typically die as a result of extracranial disease progression, whereas mortality in patients with uncontrolled BM is commonly attributable to CNS dysfunction.^11^ Optimal local control therefore remains a goal of treatment, and a thorough understanding of the effective management options for BM is essential to improve the quality of life (QOL) and overall survival (OS) in this increasingly diverse and expanding patient population. While treatment trends and paradigms have changed with the literature over the past several decades, neurosurgeons have retained a crucial role in caring for patients with BM. This review outlines the current data and recommendations for approaching the operative and adjuvant management of metastatic tumors to the brain (Table 1, Figure 2).

**Table 1:** Literature review of studies comparing treatment modalities for brain metastases

Treatment modalities				
Author (Year)	Enrollment	Study design	Median survival	Secondary outcomes
Surgery vs WBRT alone				
Patchell et al (1990)	Surgery: 25 vs WBRT: 23	RCT	Surgery: 40 wk WBRT: 15 wk *P* < .01	OS: <10% at 90 wk KPS > 70: 38 wk (surgery) vs 8 wk (WBRT)
Vecht et al (1993)	Surgery + WBRT: 32 vs WBRT alone: 31	RCT	Surgery + WBRT: 10 mo, WBRT: 6 mo, *P* = −0.04	Risk factor: extracranial metasteses
Mintz et al (1996)	Surgery + WBRT: 41 vs WBRT alone: 43	RCT	Surgery + WBRT: 5.6 mo, WBRT: 6.3 mo, no difference	KFS > 70: 32% of days (both groups, *P* = 1), Risk factors: extracranial metasteses
Rades et al (2007)	Surgery + WBRT: 99 vs WBRT alone: 96	Retrospective Cohort Study	Surgery + WBRT: 11.5 mo, WBRT: 6 mo, *P* < .01	Risk factor: extracranial metasteses, Resection improved local control and control within entire brain
Surgery + WBRT vs Surgery alone				
Patchell et al (1998)	Surgery + WBRT: 49 vs Surgery alone: 46	RCT	Surgery + WBRT: 48 wk, Surgery: 43 wk, no difference	Recurrance (*P* < .01) and neurological death (*P* < .01) less likely with radiotherapy
Surgery ± WBRT vs SRS ± WBRT				
Bindal et al (1996)	Surgery: 62 vs SRS: 31	Retrospective Cohort Study	Surgery: 16.4 mo, SRS: 7.5 mo, *P* < .01	Increased mortality after radiotherapy due to intracranial disease
Shinoura et al (2002)	Surgery + WBRT: 35 vs SRS: 28	Retrospective Cohort Study	Mean Surgery + WBRT: 34.4 mo, SRS: 8.2 mo, *P* < .01	Signifinantly longer time to recurrance (25 mo vs 7.2 mo, *P* = .02) for surgery vs SRS
Roos et al (2011)	Surgery + WBRT: 10 vs SRS: 11 + WBRT	RCT	Surgery: 2.8 mo, SRS: 6.2 mo, *P* = .2 (low accrual)	No differences in quality of life measures
Churilla et al (2018)	Surgery ± WBRT: 114 vs SRS ± WBRT: 154	RCT	N/A	Early (0‐3 mo) local control was higher after SRS, but benefit was lost with time; median follow‐up 39.9 mo
Surgery + WBRT vs SRS				
Muacevic et al (1999)	Surgery + WBRT: 228 vs SRS: 56	Retrospective cohort study	Surgery + WBRT: 68 wk, SRS: 35 wk, *P* = .19	No difference in 1‐y OS, neurological survival, and tumor control rates
Schoggl et al (2000)	Surgery ± WBRT: 66 vs SRS: 67	Retrospective cohort study	Surgery ± WBRT: 9 mo, SRS: 12 mo, *P* = .19	No difference in OS. SRS had significantly better local control rates (*P* < .05)
O'Neill et al (2003)	Surgery ± WBRT: 74 vs SRS: 23	Retrospective cohort study	One‐year OS—Surgery + WBRT: 62%, SRS: 56%, no difference	No difference in 1‐y OS. SRS had lower rate of local failure (0% vs 58%, *P* = .020)
Muacevic et al (2008)	Surgery + WBRT: 33 vs SRS: 31	RCT	Surgery + WBRT: 9.5 mo, SRS: 10.3 mo, no difference	SRS patients had more distant recurrances (*P* = .04)
Surgery + SRS vs Surgery				
Mahajan et al (2017)	Surgery + SRS: 64 vs Surgery: 68	RCT	Surgery + SRS: 17 mo vs Surgery: 18 mo, no difference	SRS after surgical resection of 1‐3 brain metastases results in significantly improved local control compared to surgery alone, local control at 1 y: Surgery + SRS: 72% vs Surgery: 43% (hazard ratio 0.46 [95% CI 0.24‐0.88]; *P* = .015).
Surgery + SRS vs SRS				
Prabhu et al (2017)	Surgery + SRS: 157 vs SRS: 66	Retrospective cohort study	Surgery + SRS: 15.2 mo, SRS: 10 mo, *P* = .01	Surgery + SRS was associated with significantly reduced local recurrance compared with SRS alone for patients with large BMs (≥4 cm^3^, 2 cm in diameter)
Lamba et al (2019)	Surgery + SRS: 19 vs SRS: 67	Retrospective cohort study	Surgery + SRS: 50.4 mo, SRS: 26.2 mo, *P* = .02	Resection, followed by cavity SRS is associated with improved survival in patients with 1 small brain metastasis and controlled or absent systemic disease
Surgery + WBRT vs Surgery + SRS				
Patel et al (2014)	Surgery + WBRT: 36 vs Surgery + SRS: 96	Retrospective cohort study	One‐year OS—Surgery + WBRT: 55%, Surgery + SRS: 56%, no difference	No difference in 1‐y OS. Higher rate of leptomeningeal spread after adjuvant SRS vs WBRT (31% vs 13%, *P* = .045)
Kepka et al (2016)	Surgery + WBRT: 30 vs Surgery + SRS: 29	RCT	Two‐year OS—Surgery + WBRT: 37%, Surgery + SRS: 10%, *P* = .046	Non‐inferiority of SRS to the tumor bed was not demonstrated in this underpowered study.
Brown et al (2017)	Surgery + WBRT: 96 vs Surgery + SRS: 98	RCT	Surgery + WBRT: 11.6 mo, Surgery + SRS: 12.2 mo, no difference	No difference in overall survival. Decline in cognitive function at 6 mo worse after WBRT (52% vs 85%, *P* < .001)
Kayama et al (2018)	Surgery + WBRT: 137 vs Surgery + SRS: 134	RCT	Surgery + WBRT: 15.6 mo, Surgery + SRS: 15.6 mo, *P* = .027 for noninferiority	Salvage SRS is noninferior to WBRT
Supplemental WBRT after SRS/Surgery				
Kocher et al (2011)	Surgery only: 79 vs Surgery + WBRT: 81 vs SRS only: 100 vs SRS + WBRT: 99	RCT	Supplemental WBRT: 10.9 min, Primary therapy only: 10.7 min, no difference	WBRT reduced 2‐y relapse at local (*P* < .05) and new sites (*P* < .03)

## 2 SURGERY FOR NEWLY DIAGNOSED BRAIN METASTASES

### 2.1 Indications and patient selection

**Figure 3:**
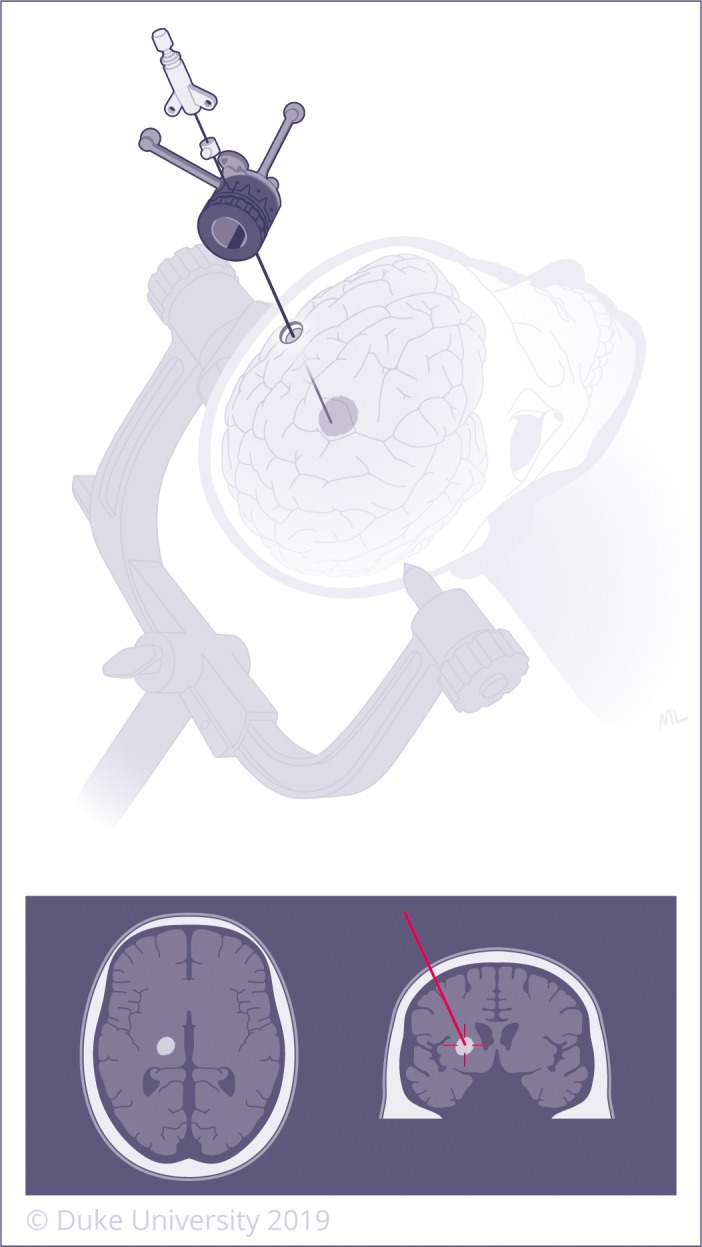
Stereotactic navigation utilizes CT or MRI images +/‐ fiducial markers to create a computerized 3‐dimensional navigation system to localize lesions deep within the brain. Biopsy can then be performed through a very small incision by introducing a needle using the navigation system. A left‐sided lesion is demonstrated on the top image and re‐demonstrated in anatomic position on the bottom image, as typically displayed by the intraoperative neuro‐navigation system

Surgery for BM involves sampling (biopsy) and/or removal (resection) of tumor tissue in the operating room. This can be done through an open craniotomy or by using minimally invasive techniques, such as stereotactic needle biopsy or laser interstitial thermal therapy (LITT, discussed in detail below). Surgery offers a number of distinct advantages over other types of therapy for BM. In 10%‐25% of cases, intracranial metastases are the only detectable evidence of a systemic primary cancer at diagnosis; for these patients, intracranial tumor sampling may offer the only means for obtaining a histologic diagnosis (Figure 3), and is thus essential for guiding further treatment.^12^, ^13^, ^14^ Patients experiencing significant neurological symptoms (such as impairments in cognition or speech, motor weakness or neglect, or seizures) attributable to mass effect, edema, or hydrocephalus benefit from resection as a means of rapid symptom resolution; it may additionally enable reduction or discontinuation of adjunct therapies such as steroids. For tumors in eloquent or difficult‐to‐access locations in the brain, whereas open resection may put the patient at high risk for postoperative neurological deficits or complications, a stereotactic biopsy may be a safer approach. Newer imaging, neuronavigation and mapping techniques, however, have mitigated much of the resection risk in many cases, and there are increasingly fewer locations that are considered truly nonoperative.

In spite of its advantages, no surgery is without risk, and surgery for BM carries an associated iatrogenic mortality of approximately 0.7%‐1.9% and morbidity of 3.9%‐6%.^9^ Add to that a typical recovery period of 4‐6 weeks, and surgeons have typically agreed that resection of BM should be limited to patients with a life expectancy greater than 3‐6 months in order to realize its benefits.^15^ The exception, of course, is when surgery may be used to palliate symptoms of mass effect in eloquent areas and restore function, even if life expectancy is somewhat short.

To maximize the probability of a favorable outcome, appropriate patient selection is critical, and much of the literature has been dedicated to prospectively identifying patient populations most likely (or least likely) to benefit from surgery. Factors such as age, Karnofsky performance status (KPS), size and number of intracranial metastases, and extent of extracranial disease play important roles in outcomes after treatment.^16^ Patient age at presentation has been found to independently correlate with clinical outcomes following resection for newly diagnosed BM and is particularly relevant in the elderly. Above age 65, comorbidities such as obesity, hypertension, diabetes, smoking, and alcohol abuse are more common,^17^, ^18^ and each of these carries independent perioperative risk. The tissue of origin also has implications for BM patients. For instance, a patient with non‐small‐cell lung cancer (NSCLC) metastatic to the brain may have undergone a lobectomy at the primary disease site, has polysystemic dysfunction or coagulopathy from metastases to the liver or viscera, or have already undergone systemic therapies, further afflicting global systemic function.^19^

Neurosurgeons must therefore work in concert with a multidisciplinary treatment team comprised of neuro‐oncologists, medical oncologists, radiation oncologists, primary care and specialist physicians, and anesthesiologists to perform a preoperative risk assessment and medical optimization to select those patients that are most likely to benefit from surgery. This approach delivers surgery within the context of a patient‐centered treatment plan, designed and executed collectively, and coordinated to maximize benefit to the patient. Lastly, thorough and pointed conversations regarding the benefits and risks of the planned treatments should be conducted between all involved parties including the treatment team, patient, and his/her family throughout the treatment course. The input of palliative care teams, when appropriate, should be sought.

Ultimately, patient care requires extrapolating relevant prognostic factors from patient populations to individual patients—a complex and nuanced task that numerous studies have attempted to streamline by creating prognostic groups from composite scores defined by shared characteristics. The first set of recommendations was proposed by the Radiation Therapy Oncology Group (RTOG) in 1997. The RTOG performed a recursive partitioning analysis (RPA) to evaluate the impact of several patient‐specific, pretreatment variables on posttreatment outcomes after radiation, with or without concomitant surgery. Of note, only 15% of the 1200 patients from three prior RTOG Phase I/III trials, approximately half of whom had multiple metastases, underwent resection and were included in their analysis. Despite these limitations, the RTOG determined that patients with a KPS ≥ 70, age < 65, “good” primary disease control, and no other extracranial metastases (defined as class 1) had a median survival of 7.1 months. Conversely, the majority of other patients (class 2) had a median survival of 4.2 months, and those with pretreatment KPS < 70 (class 3) only lived to a median of 2.3 months.^19^ This classification system was subsequently validated using historical models,^11^ and a later series suggested that RPA class 1 patients may be “more likely to [benefit] from aggressive treatment strategies”^20^ such as resection.

### 2.2 The impact of primary cancer pathology on surgical outcomes

The RPA system, despite its ease of use, reproducibility, and validation across multiple datasets, suffers from two notable shortcomings in today's treatment landscape. The first is inherent to nearly any landmark study: following two decades of treatment advances, the survival statistics are now outdated. The second is more nuanced: the scoring system does not address the impact of the primary tumor histobiology. Historic datasets have either grouped brain metastasis patients into a single category, or (in the case of clinical trials for specific cancers) excluded BM patients entirely.^21^, ^22^

Simply put, histologically distinct tumors will behave differently. Patients with BM from renal cell carcinoma and melanoma historically have had a median survival of less than 1 year, an attribute ascribed to their relative radioresistance and rapid growth.^15^, ^23^, ^24^ In contrast, the relatively slow growth and radiosensitivity of breast cancer has been tied to longer survival.^15^, ^25^, ^26^, ^27^ Compared to lung cancer metastases (particularly NSCLC), melanoma metastases are more frequently hemorrhagic on a lesion‐by‐lesion basis.^26^ Thus, while NSCLC can successfully be managed upfront with targeted radiation therapy or stereotactic radiosurgery (SRS), surgery may be the preferred initial treatment for hemorrhagic lesions.^28^ Sarcoma metastases are typically radioresistant, and *en bloc* resection of even multiple lesions provides the best prognosis for this patient population.^29^ Conversely, small cell lung cancer (SCLC), which is both relatively radiosensitive and has a propensity for local dissemination,^30^ may be best treated with whole brain radiotherapy (WBRT).^31^, ^32^

Aside from biological characteristics, the emergence of targeted therapies and immunotherapies, most of which are approved for some tumor types and not others, will have an increasingly profound effect on outcomes. Concordantly, the parameters influencing patient selection will become more esoteric: authors have advocated for patient selection by tumor‐specific genetic mutations, local disease burden, number and volume of intracranial metastases,^33^ symptomatic response to glucocorticoid therapy, serum lactate dehydrogenase levels, gender (in lung cancer), and the interval between primary tumor diagnosis and the development of BM (in breast cancer).^34^ Though each of these parameters has merit, they have not yet found a role in a validated decision‐making tool.

Recently, the graded prognostic assessment (GPA), and subsequently, the disease‐specific graded prognostic assessment (DS‐GPA) have been validated as equivalent, and possibly more precise, tools than the RPA classification system for producing survival estimates.^1^, ^16^ These scores help estimate median overall survival (OS) for patients with BM stratified by SCLC/NSCLC, renal cell carcinoma, melanoma, and breast cancer based on several factors such as age, KPS, number of intracranial metastases and presence of extracranial metastases. Both classification systems may supplant the RPA in utility as they become better‐validated through their use in patient stratification for clinical trials. However, neither has been well‐described with respect to the neurosurgical population, and a small retrospective series did not find a statistically significant relationship between preoperative GPA and postoperative survival or functional outcome.^35^ Therefore, further work is needed to best define patients that may benefit from neurosurgery.

### 2.3 Surgery for solitary brain metastasis

#### 2.3.1 Surgery + WBRT vs WBRT alone

Surgery for a single symptomatic metastasis is perhaps the least controversial topic in the literature.^36^, ^37^, ^38^, ^39^, ^40^, ^41^, ^42^ Three randomized controlled trials have been conducted comparing resection + WBRT to WBRT alone for a solitary intracranial metastasis,^38^, ^39^, ^41^ and two of the three^38^, ^39^ demonstrated a significant survival and QOL benefit for patients who underwent resection compared with those who received WBRT alone. The seminal study of this group was performed by Patchell et al in 1990, and found that the duration of functional independence (defined as KPS > 70) was significantly extended after surgery, from 1.8 to 8.8 months.^38^ Likewise, Vecht et al demonstrated that surgery was particularly beneficial for patients with absent or stable extracranial disease, while excluding patients with particularly radiosensitive subtypes from analysis, such as metastatic lymphoma, SCLC, germ‐cell tumors, leukemia, and sarcoma.^39^ The negative study in this group, published by Mintz et al in 1996, found that there was no benefit to adding surgery to WBRT (either in OS or QOL).^41^ Despite having larger patient accrual, numerous study limitations may explain the lack of observed benefit within the surgical group. The entry criteria included patients with a poorer KPS (≥50, as opposed to ≥70) and did not specify a minimum life expectancy of 6 months (as in the other two trials), resulting in 73% of enrolled patients having extracranial metastases and/or uncontrolled primary disease. There were also significant differences between the groups, with the time between diagnosis of primary tumor and metastasis being substantially shorter in the surgery group compared to the WBRT group (possibly signifying more aggressive disease), as well as a greater proportion of colorectal carcinomas and lesser proportion of breast carcinomas in the surgery group. Patients with these characteristics have been shown to have poorer survival, and consequently would be less likely to realize the benefits of surgery. Additionally, while lesion size is known to have a substantial impact on the efficacy of radiation, only one of these trials stratified data based on a maximum lesion diameter of <3 cm or ≥3 cm (ie mean 2.54 ± 1.24 cm in the surgery + WBRT group).^42^ A subsequent Cochrane review, which included a meta‐analysis of the data from the three RCTs, noted no overall survival benefit for the surgical cohorts; however, there was improvement in functionally independent survival, with no difference in neurological treatment morbidity between the two groups.^43^

After reviewing the above data, an AANS/CNS panel was created in 2010 to formulate recommendations for the operative management of newly diagnosed, single BM. This panel concluded that “class I evidence supports the use of resection + postoperative WBRT, as compared to WBRT alone, in patients with good performance status (functionally independent and spending less than 50% time in bed) and limited extracranial disease.”^44^ The panel noted insufficient evidence to make a recommendation for patients with poor performance scores or advanced systemic disease. Of note, the grading and staging of a patient's systemic disease burden is also paramount, with improved survival after surgery limited to patients with a life expectancy greater than 3‐6 months.^15^

#### 2.3.2 Surgery (±WBRT) vs SRS (±WBRT)

**Figure 4:**
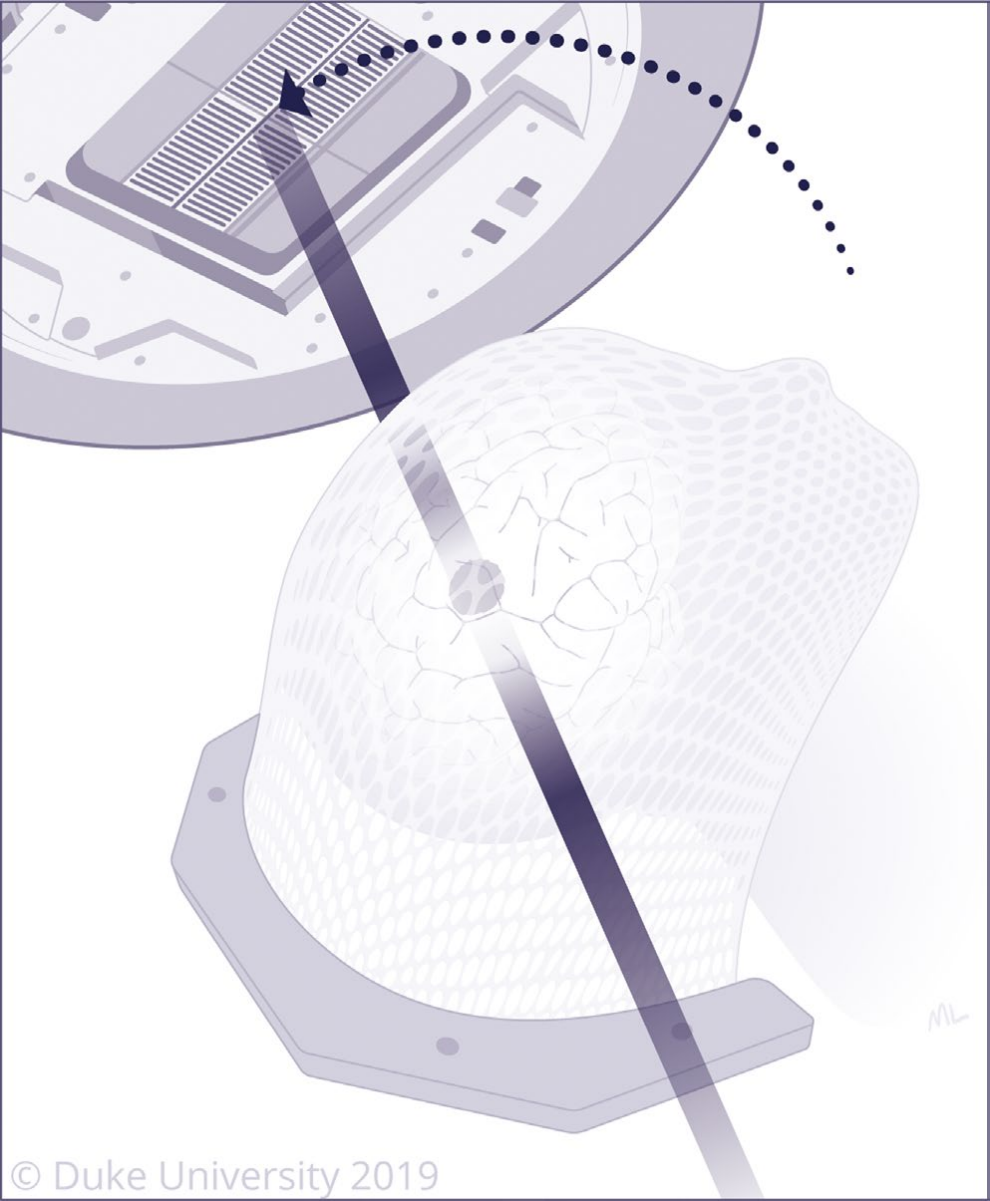
Stereotactic radiosurgery (SRS) involves delivery of precisely targeted radiation to lesions in the brain

Over the past two decades, the use of WBRT for treatment of up to four BM has fallen out of favor at many centers, calling into question the justification of surgery with literature where WBRT was the sole comparator.^45^, ^46^, ^47^, ^48^, ^49^, ^50^, ^51^, ^52^ This shift toward SRS was due to the publication of high‐quality data supporting the use of SRS over WBRT, showing comparative efficacy (sometimes even improved survival) and decreased radiation‐induced toxicity (eg cognitive decline) associated with SRS (Figure 4). While the use of SRS as a monotherapy has limitations, which include higher rates of salvage therapy, leptomeningeal disease, and distant tumor recurrences compared to WBRT,^47^, ^53^ the overall survival and cost‐effectiveness of SRS alone vs SRS + WBRT favored SRS alone in two recent studies for up to 10 metastases.^54^, ^55^ While one of the theoretical advantages of WBRT is its ability to treat multiple lesions at once, the data suggests that SRS is not limited by the number of lesions, but rather, the combined tumor volume (CTV) of the metastases, with WBRT favored only when the total tumor burden exceeds 5‐7 cm^3^.^56^, ^57^, ^58^, ^59^, ^60^, ^61^, ^62^ Indeed, Yamamoto et al showed no difference in treatment efficacy or toxicity in patients treated with SRS for 2‐9 vs >10 BM.^56^ Though CTVs are unequally distributed anatomically, and outcomes are heavily dependent on lesion location (eg a large right frontal metastasis is difficult to compare to a small pontine metastasis), the CTV of all intracranial metastatic lesions has been shown to be more predictive for outcomes after SRS than either the total number of lesions or the volume of the largest tumor.^60^

The growing volume of literature substantiating SRS as a therapeutic tool has elevated it toward direct comparison with surgery in patients with newly diagnosed BM. To that end, the efficacy of SRS vs resection (typically with adjuvant WBRT) for solitary BM has been compared in two small randomized trials^63^, ^64^ and ten retrospective cohort studies.^65^, ^66^, ^67^, ^68^, ^69^, ^70^, ^71^, ^72^, ^73^ Neither trial produced sufficient enrollment to adequately detect a survival difference between patients treated with surgery vs SRS. In the study by Muacevic et al, there was no difference in local recurrence (though the authors admitted this may be difficult to detect radiographically), as well as a slight decrease in distant brain recurrence in the surgery + WBRT arm compared to SRS.^63^ Likewise, in Ross et al's study of 11 patients treated with SRS + WBRT vs 10 patients who underwent surgery + WBRT, there was no difference seen in OS (2.8 months after surgery vs 6.2 months after SRS, *P* = .2) or quality of life measures, but was limited by low patient accrual and consequent inadequate statistical power.^64^ The retrospective studies evaluated surgery + WBRT vs SRS ± WBRT, with no clear superior treatment modality in terms of OS and duration of freedom from local recurrence. To date, there is no level 1 evidence supporting one modality over the other,^74^; the 2010 AANS/CNS panel concluded with a level 2 recommendation that both SRS and surgery “represent effective treatment strategies, resulting in relatively equal survival rates,” and a level 3 recommendation that “SRS alone may provide equivalent functional and survival outcomes compared with resection + WBRT for patients with single BM, so long as ready detection of distant site failure and salvage SRS are possible.”^44^

Though the comparative efficacy of surgery and SRS remains controversial,^75^ important patient‐specific caveats can inform treatment decisions: SRS does not provide immediate relief of mass effect, edema, or hydrocephalus, and dose reduction to accommodate a large lesion volume limits potential for local control. As a result, nearly all SRS datasets exclude tumors larger than 3‐3.5 cm and tumors causing significant mass effect (midline shift > 1 cm).^44^ One retrospective, multi‐institutional analysis by Prabhu et al in 2017 evaluated the efficacy of surgery + SRS (n = 157) vs SRS alone (n = 66) for patients with larger lesions (defined as ≥4 cm^3^; ≥2 cm in diameter), and found that surgery + SRS was associated with a significantly longer median OS (15.2 vs 10 months, *P* = .01) and reduced 1‐year local recurrence (LR) rate (36.7% vs 20.5%; *P* = .007) compared to SRS alone.^76^ Though the efficacy and safety of SRS for lesions larger than 3‐4 cm using multi‐session, hypofractionated treatment protocols has recently been investigated,^77^ surgery has remained the mainstay of initial treatment for single, large BM.^38^ (Figure 2).

### 2.4 Surgery for multiple brain metastases

As is the case with solitary BM, an understanding of the prognosis and anticipated survival is important to determining candidacy for operative management in patients with multiple metastatic brain lesions. Traditionally, patients with multiple metastases were considered to have uncontrolled primary tumors and worse prognosis, and thus were not viewed as surgical candidates. Indeed, even authors investigating surgery in the setting of multiple BM have alleged surgery in the setting of four or more BM to be impractical, noting it to be an independent predictor of poor prognosis.^78^ However, rapid advances in systemic therapy are improving outcomes for this patient population across multiple tumor types,^79^, ^80^, ^81^ generating new interest in strategies for local control.

In 2003, Pollock et al published a retrospective cohort study of 52 patients with a median of three BM, 5 of whom underwent resection alone and 16 of whom received both SRS and resection. In this study, RPA class 1 patients survived a median of 19 months, class 2 patients survived a median of 13 months, and class 3 patients survived a median of 8 months.^29^ Based on these results, the authors recommended “aggressive intervention” for RPA class 1 and 2 patients with multiple BM. Resection was recommended for patients with stable systemic disease, “good” performance status, and mass effect secondary to large lesions, based on a defined treatment algorithm.^29^ Since that time, other series have assessed the utility of RPA stratification for resection, with similar results.^82^

In an effort to compare outcomes of patients with multiple BM to those with a single BM, Bindal et al found that resection was beneficial in carefully‐selected patients, with comparatively improved survival observed after gross total resection of all lesions in patients with multiple metastases compared to age‐matched controls with a single metastasis.^83^ In 2005, Paek et al reviewed a series of patients with 2‐3 BM who underwent resection, and concluded that resection in this patient population results in similar benefits to survival and functionality compared to that of single BM, without a significant increase in perioperative complications.^74^

Overall, despite several retrospective studies demonstrating benefits to functional status and OS in this patient group,^18^, ^20^, ^29^, ^84^, ^85^, ^86^, ^87^ particularly those with three or fewer metastases,^29^, ^83^, ^88^ the role of surgery in patients with multiple BM remains controversial. This patient population is routinely excluded from surgical consideration due to a presumably shortened life expectancy,^15^, ^75^ and no randomized trials have been conducted to date to evaluate the efficacy of surgery for two or more metastases. This void in the literature was reflected in the 2010 AANS/CNS panel's conclusion that there was insufficient evidence to make a recommendation for patients with multiple BM, and further study is needed to gain clarity on this issue.^44^

## 3 THE ROLE OF ADJUVANT/NEOADJUVANT THERAPIES AND SURGERY

### 3.1 Adjuvant WBRT vs radiosurgery after surgery

Historically, WBRT has been the adjuvant therapy of choice after resection of BM.^44^, ^89^ The addition of postoperative WBRT has been shown to reduce the risk of both local and distal tumor recurrence within the brain compared to surgery alone,^90^ with an absolute risk reduction of 36% (5 [10%] of 49 vs 21 [46%] of 46, *P* < .001) and 23% (7 [14%] of 49 vs 17 [37%] of 46, *P* < .01) for local and distal tumor recurrence, respectively, as reported in Patchell et al's landmark study in 1998.^91^ However, the use of WRBT is limited by an associated increased risk of neurocognitive decline and decreased QOL.^92^ As patient survival improves, these long‐term toxicities are becoming less acceptable. Hippocampal sparing techniques offer some relief from toxicity,^93^, ^94^ and memantine has been shown to reduce cognitive decline when given concomitantly with WBRT.^95^ Overall, treatment has trended toward more targeted approaches, specifically SRS (discussed above). SRS to the resection cavity or cavities, as well as to remaining lesions within 2‐3 weeks (to allow wound healing), is now viewed as the standard of care at most academic centers, with even >20 lesions being treatable with a single plan.

Retrospective studies by Ojerholm et al and Patel et al evaluated the use of postoperative SRS to the resection bed for metastases <3 cm in diameter, and demonstrated recurrence and survival rates similar to surgery + WBRT.^47^, ^92^ Likewise, Hartford et al showed postoperative SRS without WBRT to be effective for local control, particularly for lesions with a maximum diameter of <3 cm.^96^ Another retrospective study by Smith et al demonstrated a similar 1‐year survival for patients with multiple intracranial metastases who were treated with resection followed by adjuvant SRS compared to historical data from patients receiving resection of a solitary metastasis.^97^ The overall influence on survival remains unclear, however, as other groups have reported a significant number of distant brain relapses after surgery + SRS.^98^

This initial body of literature culminated with a large randomized study (NCCTG N107C/CEC·3; NCT01372774) evaluating the efficacy of adjuvant WBRT vs SRS after resection.^99^ A total of 194 patients, 96 receiving WBRT and 98 receiving SRS, were evaluated for cognitive‐deterioration‐free and overall survival, with 64% and 55% receiving *en bloc* resection in the two groups, respectively. Patients were equally balanced across the two arms for solitary BM (77%) and for resection of solitary metastases ≤ and >3 cm. Two‐thirds of each cohort had primary lung tumors. No difference in overall survival was observed across the two arms after postoperative SRS vs WBRT. Patients who received adjuvant WBRT, however, had a worse cognitive‐deterioration‐free survival (HR = 0.47). At 6 months, cognitive deterioration was less frequent in the SRS group than in the WBRT group (52% vs 85%). Local surgical bed and distant brain control were similar in the two groups, though several confounders made it difficult to interpret SRS as inferior (noncentral review of putative pseudoprogression and more subtotal resections in the SRS cohort being the primary limitations). Ultimately, the study concluded that SRS was not inferior to WBRT for patient survival. Although prior studies suggest WRBT may offer improved distant tumor control, these results imply that adjuvant SRS should indeed supplant adjuvant WBRT as the standard of care for patients with BM due to equivalent survival, better preservation of cognitive function and QOL, and less toxicity than WBRT.

### 3.2 The role of neoadjuvant SRS

One of the challenges limiting the use of adjuvant SRS is the possibility of pachy‐ or leptomeningeal seeding after open surgery,^100^ particularly if the tumor location near eloquent cortex limits the surgeon's technical ability to perform the resection *en bloc*. A recent study in JAMA by Cagney et al found that surgery for newly diagnosed BM was associated with pachymeningeal seeding, but not leptomeningeal disease, with pachymeningeal seeding occurring in 8.4% (36 of 428) of operations. Of the 36 patients with seeding, 27 (72%) died as a result of progressive pachymeningeal disease. Furthermore, a higher incidence of pachymeningeal seeding was found after resection of previously irradiated vs unirradiated metastases (HR, 2.39; 95% CI, 1.25‐4.57; *P* = .008).^101^ In an attempt to overcome the limitations of postoperative SRS, neoadjuvant SRS, delivered immediately prior to resection, has been investigated as an alternative. The rationale for this treatment timeline is presumed reductions in the risk of pachy‐ and/or leptomeningeal disease (due to sterilization of tumor cells prior to spillage at the time of surgery), as well as radiation necrosis (RN, due to lower irradiated volumes without the cavity margin expansion that is necessary postoperatively). Asher et al reported an overall survival rate of 77.8% and 60.0% at 6 and 12 months, respectively, with neoadjuvant SRS, as well as local control rates of 97.8% and 85.6%.^102^ Patel et al performed a retrospective analysis of patients who underwent either preoperative SRS or postoperative SRS, finding a lower incidence of leptomeningeal disease with preoperative SRS.^103^ These two studies were then combined into an expanded and updated analysis, which reaffirmed these results and concluded that single‐fraction neoadjuvant SRS confers excellent local control with very low risk of RN or leptomeningeal disease (4.8% and 4.3%, respectively, at 2 years).^104^ Given this favorable early data, multiple prospective trials investigating the utility of neoadjuvant SRS (NCT01891318, NCT03163368, NCT03368625) are currently accruing.

### 3.3 Adjuvant immunotherapy after surgery/SRS

The role of immunotherapy for patients with BM, particularly its efficacy as an adjunct to surgery, is largely unknown. The limited number of available studies is summarized in an excellent and recent review by Kamath and Kumthekar.^105^ One notable 2019 single‐institutional retrospective analysis compared the impact of immunotherapy (CTLA‐4 ± PD‐1 or PD‐1 inhibitors) and targeted therapy (BRAF ± MEK inhibitors) alone or in combination with surgery and/or radiotherapy in patients with melanoma BM. Over a median follow‐up period of 25 months in 163 patients with melanoma metastases, the median OS for patients receiving immunotherapy vs targeted or chemotherapy (carboplatin/paclitaxel, dacarbazine or temozolomide) alone was 13 and 7 months, respectively. When immunotherapy, targeted therapy, and chemotherapy were used in combination with surgery and/or SRS, the median OS was 25, 14, and 11 months, respectively. In an effort to reliably evaluate the comparable efficacy of various treatment options, the authors first defined the “dominant” therapy as the modality that was administered for the longest period of time or that achieved the best disease control or response. The “dominant” systemic therapy options were then stratified by four groups: immunotherapy (IT), targeted therapy (TT), chemotherapy (CT) and no systemic therapy. Lastly, each “dominant” therapy was analyzed with combination of surgery and/or SRS (S/RS) across the following six groups: S/RS + IT, S/RS + TT, S/RS + CT, S/RS + no systemic therapy, WBRT ± systemic therapy and no radiotherapy (with or without systemic treatment). As noted above, the combination of S/RS + IT had the longest median OS at 25 months, followed by S/RS + TT at 14 months, and S/RS + CT at 11 months, compared to only 4 months with S/RS without any systemic therapy. Based on these results, the authors concluded that new systemic therapies, especially immunotherapies, improve the OS of patients with melanoma BM, particularly when combined with S/RS. While this study did not specifically identify which patients received surgery vs SRS, the results support further work on the utility of newer systemic therapies in patients with BM.^106^ Of further importance will be elucidating the optimal timing of immunotherapy with respect to surgery and SRS, a concept that has only recently begun appearing in the literature.

## 4 SURGERY FOR RECURRENT BRAIN METASTASES

### 4.1 Surgery for previously resected brain metastases

Secondary resection of recurrent BM has been shown to prolong survival in RPA class I patients^11^, ^107^, ^108^ predominantly when either the time interval from primary diagnosis to initial BM or the duration between first metastasis and metastatic recurrence is relatively short.^109^ A study of 147 cases of metastatic melanoma found that craniotomy for recurrence was associated with a significantly increased survival. Nearly 16% (n = 24) of patients underwent reoperation for recurrent intracranial disease following initial treatment, and 8% (n = 2) of this cohort became long‐term survivors.^110^ Of note, half of the patients died due to complications of systemic, not intracranial disease. Although resection of recurrent intracranial tumors was associated with improved survival compared with those who were managed conservatively, this result should be interpreted cautiously due to the small sample size and selection bias toward patients able to endure a subsequent surgery (bias of indication). Operating on previously resected lesions also bears technical challenges. For example, scarring and adhesions from prior operations can make dissection arduous and mapping eloquent locations difficult to achieve. Associated risks include prolonged operative time, hemorrhage, wound dehiscence, and infection. Because of these concerns and the lack of randomized studies, the 2010 AANS/CNS panel on retreatment in metastatic disease concluded that “there is insufficient evidence to make definitive treatment recommendations in patients with progressive BM, recurrence, or progression at original vs nonoriginal site.”^111^ However, despite limited evidence, secondary resection can be considered for accessible, symptomatic lesions with radiographic or histologic evidence of tumor recurrence, and reoperation may indeed be beneficial provided a sufficiently low morbidity rate.^112^ (Figure 2).

### 4.2 Surgery for previously irradiated brain metastases

Radiographic progression in the aftermath of radiation typically portends one of two diagnoses: recurrent/progressive disease or radiation necrosis (RN). RN is a delayed complication of irreversible, peritumoral tissue injury that results from a combination of endothelial cell apoptosis,^113^ peritumoral inflammation,^114^, ^115^, ^116^ blood–brain barrier disruption,^117^ and chronic tissue hypoxia.^118^ It is admittedly a misnomer, as it is frequently a proliferative and expansile inflammatory process that has variable response to first‐line anti‐inflammatory treatments such as dexamethasone. The authors advocate relabeling the condition as Radiation‐Induced Delayed Inflammatory Response (RIDIR) and will refer to it as such throughout the remainder of this review. There is no imaging modality that can reliably differentiate between RIDIR and true progression; however, the temporal relationship to radiation may provide a clue. Our group recently reviewed the histopathological diagnosis of 35 patients with prior radiation undergoing stereotactic biopsy for radiographic progression. When biopsy was performed within 9 months from radiation treatment of the index lesion, pathology revealed a near 50/50 split between recurrent/progressive disease and RIDIR. Beyond 9 months, however, >90% of diagnoses were RIDIR.^119^ Ultimately, pathology is required for unequivocal diagnosis, and biopsy remains the gold standard to permit diagnosis and treatment (Figure 3). Due to sampling error inherent to needle biopsy, open biopsy or resection can provide advantages of more accurate and thorough tissue diagnosis, as well as added possibility of local control and symptom relief. In contrast to RIDIR, recurrent or progressive disease may require additional salvage radiation (this is contraindicated for RIDIR), and surgery may play a role for both.

#### 4.2.1 Open surgery for recurrent/progressive disease after radiation treatment

There is currently limited guidance regarding resection after SRS failure. Vecil et al evaluated outcomes of 61 patients with recurrent BM who underwent resection after failed SRS.^108^ The median overall survival was 11.1 months, with 25% of patients surviving 2 or more years. Not surprisingly, patients with a favorable RPA (class 1 or 2) and those with additional lesions that were aggressively treated with focal modalities (surgery or SRS, compared to nonfocal modalities such as WBRT and chemotherapy) were most likely to benefit from resection. Resection also facilitated tapering of steroid therapy in this study with less than 5% of patients requiring long‐term steroids after surgery, compared to the 40% who were steroid‐dependent for 12 consecutive weeks prior to surgery. This study therefore supports the benefit of secondary resection following SRS failure as a means to control both symptoms and disease in those with favorable prognostic markers.

Local control after resection for SRS failure may depend on the interval between SRS and resection. One retrospective study of 14 patients evaluated the benefit of post‐SRS resection for recurrent metastasis, finding that a shorter time interval between SRS and resection was associated with a lower frequency of local failure—indirectly supporting the role of neoadjuvant SRS, albeit not in treatment‐naïve patients. In patients who underwent resection within three months after SRS, there was no local recurrence, suggesting that neoadjuvant SRS is feasible in certain patients. However, due to the limited sample size, investigators were unable to detect a difference in survival if surgery was offered within this time frame.^120^ Given the retrospective nature of this study and the observed benefit in subsets of these patients, further investigation in this area is warranted.

#### 4.2.2 LITT for recurrent/progressive disease after radiation treatment

**Figure 5:**
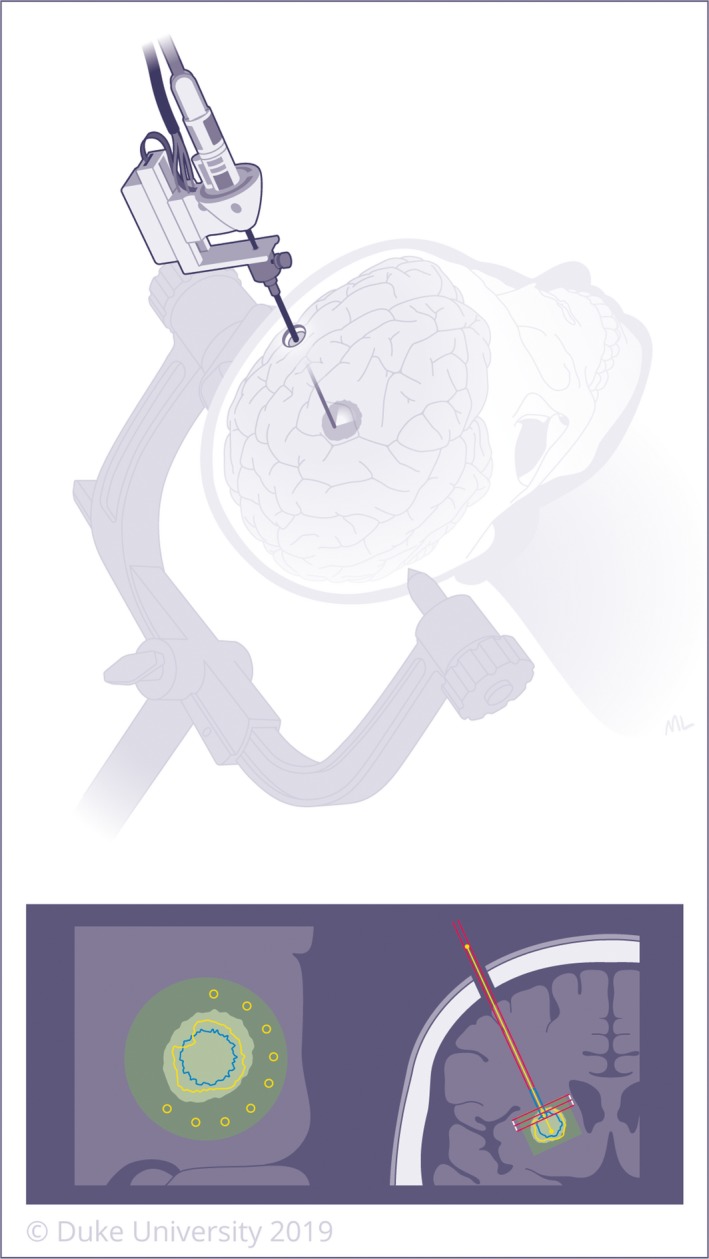
Laser interstitial thermal therapy (LITT) involves inserting a laser probe through a small hole using the same trajectory from the stereotactic biopsy. The laser then induces thermal energy, leading to cellular damage and protein denaturation. Propagation of heat during LITT is monitored using MR thermography. A left‐sided lesion is demonstrated on the top image and re‐demonstrated in anatomic position on the bottom image, as typically displayed by the intraoperative neuro‐navigation system

Among the novel strategies emerging as an alternative to surgery for recurrent BM is laser interstitial thermal therapy (LITT) (Figure 5). LITT is a minimally invasive surgical intervention that utilizes a laser probe inserted through a burr hole to provide optical radiation that focally delivers thermal energy to induce cellular damage (Figure 5). The procedure is guided by MRI thermography, and the intended increase in tissue temperature initiates a cascade of events including protein denaturation, enzyme expression, membrane disruption, vascular sclerosis, and coagulative necrosis.^121^ Advantages of LITT include safe access to lesions for which open resection is unappealing, obviating wound‐healing or intracranial scarring issues that complicate open surgery in patients with lesion recurrence after surgery or radiation, or providing an alternative to surgery for patients too medically frail for a craniotomy. Relative limitations to LITT include superficial or periventricular lesions (due to cerebrospinal fluid acting as a heat sink), although these are not absolute. Additionally, the use of LITT may be limited to lesions with a maximum diameter of <3 cm.^122^ These attributes make LITT best‐suited for deep or recurrent metastases.

While the benefit of this therapy has been extensively demonstrated for the ablation of lesional epilepsy, RIDIR, and gliomas located in deep‐seated or eloquent parenchyma, data regarding its use as an adjuvant treatment modality for recurrent BM remains sparse. Carpentier et al presented four patients who received LITT for six recurrent, focal BM, and found it be to a safe and effective treatment option, with no evidence of recurrence in any of their treated lesions within three months after treatment.^123^ The study, however, was not designed to demonstrate treatment efficacy, and follow‐up is greatly limited. Ali et al showed LITT to abort radiographic progression in 23 patients with recurrent BM after SRS when coverage of the lesion exceeded 80%.^124^ These results underscore the importance of complete ablation to achieve local control. Inherent to this concept is the size limitation for LITT, not only due to tissue volume, but also potential morbidity associated with LITT‐induced edema in larger lesions. Therefore, the authors suggested using adjuvant hypofractionated radiotherapy (ie 5 fractions of 5 Gy) for cases where <80% of the lesion is ablated with LITT.^124^ Despite these shortcomings, LITT may be an effective treatment option for up to 20% of patients who fail radiation therapy, as it does not preclude patients from receiving any other treatment modality at a later date (ie further SRS), including additional LITT. Of note, no prospective, randomized trial has yet been conducted to demonstrate the long‐term efficacy of LITT for recurrent metastatic brain lesions or RIDIR.

#### 4.2.3 Surgery for Radiation Necrosis/Radiation‐Induced Delayed Inflammatory Response (RIDIR)

RIDIR typically manifests in a delayed fashion, appearing 3‐9 months after initiation of therapy^125^, ^126^ and escalating in its probability as an explanation for radiographic progression after nine months.^119^ As mentioned previously, the diagnosis and management of RIDIR after SRS for BM is particularly challenging given that both RIDIR and recurrent tumor display similar characteristics on imaging, including contrast enhancement.^127^ Despite the use of several radiographic techniques such as perfusion MRI, DWI, MR spectroscopy and PET CT,^128^ tissue biopsy is required for definitive diagnosis (Figure 3).^119^ Moreover, the true incidence of RIDIR is unknown due to the difficulty with accurate identification and lack of standard diagnostic criteria. While the average reported incidence of RIDIR after SRS is approximately 9%‐14%,^129^, ^130^ with some studies reporting frequencies as high as 24%‐82%,^131^, ^132^ rates are up to three times higher in patients receiving concurrent chemotherapy.^133^, ^134^, ^135^ Furthermore, the incidence of RIDIR is increasing with the more frequent use of SRS and multimodal treatment, particularly immunotherapy.^136^ To complicate matters further, cases of pure RIDIR without any recurrent tumor present are rare, and most studies do not distinguish between isolated and mixed cases.^137^ While several different treatment modalities have been employed for the treatment of symptomatic RIDIR, including corticosteroids, hyperbaric oxygen, Trental/Vitamin E, anticoagulation, and bevacizumab,^136^ biopsy with subsequent LITT or resection is often necessary to accurately differentiate it from recurrent tumor,^5^, ^75^ as well as to treat the associated mass effect or edema.^119^, ^122^, ^128^, ^136^ Both LITT and resection offer the advantage of early diagnosis from an accompanying biopsy, and both can likewise be employed effectively regardless of biopsy result, with adjuvant radiotherapy reserved for recurrent/progressive disease.

## 5 OPERATIVE, PERIOPERATIVE AND OTHER MANAGEMENT CONSIDERATIONS

### 5.1 Preoperative and intraoperative planning

Once a patient is determined to be a surgical candidate, extensive preoperative planning by the surgeon and ancillary operative staff, in concert with the patient's medical oncology and radiation oncology team, is essential to safely and effectively remove BM. Considerations such as lesion size, tumor location and surrounding structures, approach to the lesion, and operative technique are paramount.^9^, ^138^ The structural and functional imaging characteristics of the metastasis and surrounding parenchyma can be examined via high‐resolution MRI.^3^, ^139^ Fortunately, modern technical advancements in functional MRI, diffusion tensor imaging (DTI), neuronavigation software, neurocognitive monitoring, awake craniotomy, and microsurgical tools have greatly aided neurosurgeons in achieving gross total resection via smaller craniotomies, shorter operative times, and with a reduction in surgical complications.^139^, ^140^, ^141^, ^142^ These invaluable techniques and technologies have also allowed experienced surgeons to safely operate in eloquent brain areas. In general, neurosurgical patients receive a thin‐slice, preoperative MRI (or CT in patients in whom an MRI cannot be performed, eg those with a noncompatible pacemaker) for use in intraoperative, stereotactic image guidance. For lesions located in eloquent areas, neuromonitoring can be employed, either awake or asleep for motor mapping, and awake for speech mapping. Functional MRI and/or DTI imaging are used on a case‐by‐case basis to permit optimal surgical planning to avoid speech centers in the brain and critical white matter fiber tracks, respectively.

Special consideration should be given to cerebellar metastases given the limited size of the posterior fossa and proximity to the brainstem and critical CSF flow pathways. In 2009, Yoshida and Takahashi reported a substantial improvement in survival after resection of cerebellar metastases, with a median survival of 35.5 months in patients treated with resection + adjuvant radiation, 20.5 months in patients treated with resection alone, 9.1 months after SRS alone, and 6.5 months after WBRT alone.^143^ Since larger posterior fossa metastases confer a comparatively high risk of obstructive hydrocephalus and brainstem compression, surgery may often be the most viable initial treatment option to avoid potentially life‐threatening radiation‐induced edema and mass effect.

### 5.2 Impact of extent of resection

Prior studies have shown that the extent of resection is one of the most important factors in determining outcomes after surgery for metastatic brain lesions, with *en bloc* resection resulting in better local tumor control and decreased recurrence compared to subtotal and/or piecemeal resection.^9^, ^138^, ^144^ Despite radiographic confirmation of gross total resection on immediate postoperative MRI, local recurrence occurs in approximately 10%‐34% of patients at 1 year postresection and adjuvant radiotherapy, depending on the primary pathology.^9^ This may be due to the fact that metastases may produce up to 1‐3 mm of clinically and radiographically undetectable tissue infiltration, as has been confirmed in neuropathological specimens.^145^, ^146^ In their landmark 2006 study, Baumert et al showed that approximately 63% of BM exhibited infiltrative growth beyond the border of the grossly and radiographically visible tumor mass.^145^ Based on this, some surgeons have proposed performing “microscopic total resection”—tumor removal with an additional margin of approximately 5 mm into normal‐appearing surrounding brain parenchyma when anatomically feasible using an ultrasonic aspirator. With this technique, Yoo et al reported better local tumor control compared to traditional gross total resection, with 1 and 2‐year local recurrence rates of 29.1% and 29.1% vs 58.6% and 63.2%, respectively.^147^ Unfortunately, this method is not feasible in or near eloquent areas, limiting this technique to the nondominant frontal lobe, nondominant temporal lobe, or cerebellum.

### 5.3 The role of surgery in leptomeningeal disease

With the advances in systemic cancer treatment and prolonged patient survival, the incidence of leptomeningeal disease is rising.^148^ Certain metastatic cancers hold a predilection for leptomeningeal spread over others, with breast cancer carrying the highest risk.^100^ No surgery directly treats leptomeningeal disease; however, as an alternative to serial lumbar punctures for drug delivery into the lumbar cistern, surgeons can place an Ommaya reservoir (a silicone reservoir attached to an intraventricular catheter) for delivery of intrathecal chemotherapy. Ommaya insertion has higher upfront risks as a surgical procedure, but allows for easier and less painful access to the cerebrospinal fluid space for chemotherapy administration in the outpatient setting while allowing for CSF sampling directly from the ventricle.^148^

It is important to consider the risk of surgical “spillage” and leptomeningeal seeding when operating on large lesions that limit *en bloc* resection, particularly in lesions abutting major CSF pathways—such as in the posterior fossa or within the ventricular system.^138^, ^144^, ^149^ In a recent study of 379 patients with posterior fossa metastasis treated with either resection or SRS, the rate of postoperative leptomeningeal spread was significantly lower after *en bloc* resection (5.6%) than piecemeal resection (13.8%).^149^ Even in spite of careful operative technique, microscopic spillage can ensue with any surgical intervention. Neoadjuvant SRS (as discussed above) may have a role in decreasing the dissemination of viable tumor cells throughout the CNS,^102^, ^103^, ^104^ but there is limited evidence in this area.

### 5.4 Use of anticonvulsants in patients with brain metastases

The role of perioperative antiepileptic drugs (AED) has been considered in patients with cerebral metastases. Metastatic brain tumors overall may be less likely to cause seizures compared to primary intracranial malignancies.^150^, ^151^ Of BM, lung cancer (both non‐small cell and small cell) is most commonly associated with seizure, followed by metastases from the breast, skin and colon.^14^ The predominant causes of seizures in the context of BM come from offending biologic processes such as toxic systemic chemotherapy, paraneoplastic limbic encephalitis, metabolic disorders, CNS infections,^150^ and SRS‐induced RIDIR,^152^ for which a variety of anti‐epileptic drug regimens have demonstrated formidable seizure control. As patients continue to live longer with intracranial metastasis, it has become increasingly important to distinguish and control for seizures that result from treatment effect and adjunct medications, as opposed to those that stem from the natural history of the disease.

To date, the only randomized trial to examine the role of prophylactic AED in patients with newly diagnosed brain tumors showed no difference in seizure incidence in the metastatic tumor subgroup.^153^ Likewise, in a retrospective cohort study, as well as in a later randomized trial of AED use after surgery, there was no reduction in the incidence of seizures in intra‐axial brain tumors (including metastases) with the use of postoperative AED.^154^, ^155^ Ansari et al evaluated 202 patients with intra‐axial brain tumors, and found no difference in postoperative seizure rates for glioblastoma vs other tumor types (including metastases). Similarly, Wu et al reported a randomized study of 123 patients (77 with metastases and 46 with gliomas) and found no significant difference in seizure rates for patients who did or did not receive postoperative AED, in both the metastasis (15% vs 13%, *P* = 1.00) and the glioma group (39% vs 26%, *P* = .53).^155^ In light of this data, the 2010 AANS/CNS panel concluded that routine, prophylactic AED (either pre‐ or postoperatively) are not recommended for patients with BM.^156^, ^157^ Therefore, anticonvulsants are typically reserved for patients who present with seizure, or have a pre‐existing indication for AED use.

### 5.5 Use of prophylactic and therapeutic anticoagulation in patients with brain metastases

Patients with a known malignancy carry an intrinsic four‐ to seven‐fold higher risk of venous thromboembolism (VTE), and cancer is an independent risk factor for VTE. Within this hypercoagulable population, patients with brain tumors have a particularly high incidence of VTE.^15^ Early diagnosis and treatment is an absolute necessity. If left untreated, nearly half of patients with symptomatic, proximal deep venous thromboses (DVT) will develop clinically evident pulmonary emboli (PE),^158^ which carry mortality rates of 17% to 34%.^159^, ^160^ While mechanical approaches, such as vena cava filters or (in rare circumstances) mechanical thrombectomy, may be the only option in a recently postoperative patient to prevent or treat a life‐threatening PE, these interventions do nothing to address the initial clot. Additionally, these mechanical approaches have high complication and treatment failure rates in patients with intracranial malignancies.^161^ Thus, initiation of systemic anticoagulation is almost invariably necessary when deemed sufficiently safe by the surgeon.

Warfarin has remained a common anticoagulant in brain tumor patients, but its therapy is fraught with the risk of supratherapeutic dosing and iatrogenic intracranial hemorrhage (ICH), especially given its known interactions with other recommended prophylactic medications (ie proton pump inhibitors, anticonvulsants, antibiotic prophylaxis). When compared to patients without malignancy, cancer patients on warfarin have increased rates of both recurrent VTE and pathologic bleeding.^162^, ^163^, ^164^ Thus, alternative anticoagulants, such as low molecular weight heparin (LMWH) and heparin derivatives, including: dalteparin, enoxaparin, and tinzaparin are being used effectively with increasing frequency as initial therapy for symptomatic, proximal DVT^165^, ^166^ and PE.^167^ The CLOT trial (Randomized Comparison of Low Molecular Weight Heparin vs Oral Anticoagulant Therapy for the Prevention of Recurrent Venous Thromboembolism in Patients With Cancer), which included 27 patients with brain tumors, was a seminal study on therapeutic anticoagulation for malignant VTE, demonstrating that cancer patients with VTE who received long‐term dalteparin were 50% less likely to develop recurrent VTE than patients treated with warfarin.^168^ As a routine practice for most cancers, patients with known malignancy and documented VTE are initiated on the subsequent derivative enoxaparin 1mg/kg/twice daily until ostensibly cancer‐free. Furthermore, newer agents, such as direct factor Xa inhibitors, are being utilized with increased frequency since the favorable results of the 2018 Edoxaban study, which showed noninferiority to LMWH (dalteparin) in patients with cancer‐associated VTE.^169^ As such, these agents are starting to replace routine LMWH in patients with metastatic disease. Based on the increase risk of intracranial hemorrhage in the intraoperative and postoperative period, as well as the current limited availability of reversal agents, these agents are typically held for at least 3‐5 days prior to surgery depending on their respective half‐lives.

To date, no head‐to‐head trial has been performed comparing LMWH to warfarin to reduce the rate of VTE in patients with intracranial malignancies alone. Such a study could be confounded by the propensity for certain tumors to exert an endogenous bleeding risk, the fragile state of thrombocytopenia in certain patients, and the bleeding risk from recent neurosurgery.^170^ Of the metastatic cancers, those with the highest rates of spontaneous ICH include thyroid cancer, melanoma (40% to 50%), renal cell carcinoma (up to 70%), and choriocarcinoma (almost all cases).^170^, ^171^, ^172^, ^173^ In contrast, metastases from breast and lung cancer, which together account for nearly 70% of all BM, have spontaneous hemorrhage rates <1% and 5%, respectively.^174^ Given the proclivity for certain tumors to develop spontaneous ICH in the absence of any anticoagulation, there is apprehension for subjecting patients to further bleeding risk, which may be limiting enthusiasm for prospective clinical trials testing various agents.

A retrospective study of 293 patients with BM on therapeutic enoxaparin that were matched with nonanticoagulated controls showed that the risk of ICH was four‐fold higher (adjusted HR 3.98, *P* < .001) in patients with melanoma or renal cell carcinoma (n = 60) than in those with lung cancer (n = 153). A second retrospective study evaluated the relative risk of spontaneous ICH in melanoma BM patients on therapeutic enoxaparin, finding that there was no significantly increased risk with this type of tumor. Specifically, there was no difference in bleeding risk between patients with a maximum metastatic foci diameter of <1 cm (n = 24) vs >1 cm (n = 41), although very few patients in this cohort had large foci (3 pts with 1‐2 cm; 12 pts, 2‐3 cm; 3 pts,> 3 cm). Furthermore, the 41 patients with metastases >1 cm were treated with a reduced dose of enoxaparin (1 mg/kg once daily) and had effectively resolved PE in 15 of 15 evaluated patients, suggesting that this is a reasonable regimen to consider for such patients.^175^

While the role of therapeutic anticoagulation in patients with BM remains controversial, in perioperative patients who have never had VTE, the use of pneumatic compression stockings and early initiation of VTE chemoprophylaxis (within 24‐48 hours postoperatively) has been recommended.^15^

### 5.6 Use of perioperative anti‐VEGF agents in patients with brain metastases

While the literature specific to chemotherapy and BM remains sparse, several agents, especially those that inhibit the actions of vascular endothelial growth factor (VEGF), have been shown to increase the risk of hemorrhage and other complications in metastatic brain tumors as well as other primary CNS tumors (eg glioblastoma). As such, the perioperative management of patients receiving various systemic therapies is an important consideration for resection of BM.

VEGF inhibitors may be used as monotherapy or in combination with other chemotherapeutic agents, and they have been approved for treatment of many tumor types, including colorectal, NSCLC, breast, glioblastoma, renal cell carcinoma, hepatocellular carcinoma, gastrointestinal stromal tumor, pancreatic neuroendocrine tumors, and medullary thyroid cancers.^176^ Disruption of VEGF signaling, either by blocking VEGF directly or the tyrosine kinase receptor at which it exerts its effects, has been shown to slow tumor progression by preventing new vessel formation. While anti‐angiogenic agents such as these offer a favorable toxicity profile compared to traditional chemotherapeutic agents, they may be associated with an increased risk of bleeding.

Bevacizumab is a recombinant IgG1 monoclonal antibody against VEGF that is currently approved for the treatment of metastatic breast cancer, colon cancer, and NSCLC, although it is also used off‐label for the treatment of RIDIR.^177^, ^178^, ^179^, ^180^ The bleeding risks associated with bevacizumab and other anti‐VEGF agents range in frequency and severity, including epistaxis, hemoptysis, hematemesis, GI bleeding, vaginal bleeding, and intracranial hemorrhage.^181^ The mechanism behind bleeding events is likely multifactorial, but is primarily thought to be due to increased vascular fragility and rupture of newly formed vessels.^182^ One commonly referenced reported case describes a single major cerebrovascular bleeding event that occurred 14 days following a single dose of bevacizumab (3 mg/kg) in a patient with a previously unrecognized BM.^183^ As a result, patients with BM are thought to be particularly susceptible to bleeding events, especially at the site of brain lesions, and are therefore typically excluded from most clinical trials.

While these adverse events are serious, there is a growing body of evidence that suggests intracranial bleeding is a rare adverse event that should not preclude patients with brain tumors from participation in bevacizumab clinical trials. A review by Sandler et al highlights three studies that support the low incidence of intracranial bleeding events among patients with BM receiving anti‐VEGF therapy.^181^ Likewise, a retrospective review of 57 published trials with 10 598 total patients by Carden et al concluded that the rate of intracranial bleeding among patients treated with either bevacizumab, sorafenib or sunitinib was negligible, even in the presence of BM, and suggests that patients with controlled brain tumors should not be excluded from future clinical trials for anti‐VEGF therapy.^184^ Of note, however, the reviewed trials did not report the incidence of metastatic brain tumors, and 76% of the trials listed CNS metastases as an exclusion criterion.

In another study supporting the safety of VEGF inhibitors, Oh et al conducted a pharmacy database review of 149 421 total patients, of which 6674 patients (4.5%) had a brain tumor (primary or metastatic).^185^ Of the 1360 bevacizumab‐treated patients, 179 (13.2%) had BM. They concluded that the prevalence of intracranial bleeding in patients with brain tumors was similar among those treated with bevacizumab (4.5%, n = 8) and those who were not (4.4%, n = 286, *P* = .887). The prevalence of cerebrovascular infarct was slightly higher among bevacizumab‐treated patients (1.7%, n = 3) compared to control (0.5%, n = 32), but this heightened risk was not statistically significant (*P* = .095).^185^ Moreover, Besse et al conducted a retrospective analysis of 17 trials of bevacizumab therapy; 639 patients had BM, of which 131 were known and pretreated, and 508 were undiagnosed at the time of study entry. All 131 of patients with pretreated BM and 412 of the undiagnosed patients were then treated with bevacizumab. Results indicated an overall low rate of intracranial bleeding events: 0.8% in those with pretreated BM, and 0.9% (large, open label single‐arm safety studies) and 3.3% (randomized controlled trials) in those who were unknown or later developed BM. This is compared to a 1.0% rate of intracranial bleeding events among patients who did not receive bevacizumab.^186^

Numerous clinical trials have strongly supported the safety, effectiveness, and survival advantage associated with bevacizumab.^179^, ^187^, ^188^ However, in the setting of resection, there are still important complications to its use that are relevant to surgeons. To illustrate, Gordon et al published a review of bevacizumab and its effect on surgical wound healing, including increased risk of dehiscence, ecchymosis, surgical site hemorrhage, and wound infection.^177^ This review refers to a randomized phase II trial by Kabbinavar et al, which found that patients who had been treated with bevacizumab had a significantly increased bleeding risk (59%) compared to patients who had chemotherapy alone (11%).^178^ In addition, thrombotic events (eg deep venous thrombosis, pulmonary embolism, catheter‐related thrombosis, transient ischemic events, and cerebrovascular accidents) were more frequent and more severe in the surgical group as compared to chemotherapy alone.^178^ A pooled analysis of 5 randomized controlled trials encompassing 1745 patients randomly allocated to chemotherapy vs chemotherapy + bevacizumab for treatment of metastatic carcinomas (colorectal, breast, and non‐small cell lung) showed that the addition of bevacizumab was associated with a twofold increase in rate of arterial thromboembolic events (ATE) (3.8% vs 1.7%, *P* = .031).^189^ Scappaticci et al showed that the risk of ATE was particularly higher among elderly patients (age ≥ 65) or those with past history of ATE, with as many of 17.9% of patients with both risk factors developing ATE with bevacizumab therapy.^190^ However, these studies did not specifically focus on patients with CNS malignancies. This heightened risk of bleeding and/or thrombotic complications may be of important consequence when treating highly vascular intracranial tumors with bevacizumab. This is especially of concern in the setting of surgery, since VEGF has been shown to play an important role in wound healing and undergoes upregulation for at least one week following surgery.^191^

The literature also suggests that some tyrosine‐kinase inhibitors, such as sunitinib and sorafenib, have an associated risk of bleeding; thus, patients with known CNS metastases have also been generally excluded from clinical trials investigating these agents.^181^ These agents target the VEGF and platelet‐derived growth factor receptors (PDGFR), among others, to inhibit angiogenesis and halt tumor growth, invasion, and metastasis. They have been approved for use in various malignant cancers, including renal cell carcinoma, hepatocellular carcinoma, NSCLC, pancreatic neuroendocrine tumors, and gastrointestinal stromal tumors.^181^, ^192^, ^193^ A systemic review and meta‐analysis of clinical trials by Je et al investigated the reported bleeding events in a total of 6779 patients treated with either sunitinib or sorafenib for a variety of cancers and found a two‐times increased risk of bleeding events in patients treated with either of these agents. The incidence of all bleeding events was found to be 16.7% (95% CI 12.7‐21.5), while that of high‐grade bleeding events was 2.4% (95% CI 1.6‐3.9).^192^ In addition, Pouessel et al found an increased bleeding risk with the use of sunitinib and sorafenib—specifically, a high incidence of fatal intracerebral hemorrhage in patients with BM secondary to renal cell carcinoma.^193^ Of the 7 patients with known BM (solitary or multiple) who were treated with sunitinib or sorafenib, 4 developed acute, massive, and fatal intracerebral hemorrhage between days 2 and 14 from treatment onset.^193^

While newer immunotherapeutic agents are generally not considered to present an operative risk, patients who experience immune related adverse events on these agents often require specialized management.^194^ This may include long‐term high dose steroids, and other immunosuppressive agents that may impact wound healing and infection risk. Additionally, patients with endocrinopathies such as hypophysitis or adrenal insufficiency will likely need stress dose steroids peri‐operatively, and close coordination with an endocrinologist is advised. The timing and dosages for tapering steroids are typically at the discretion of the surgeon and other members of the multidisciplinary care team. A common practice is to taper steroids to a low dose or off (maintenance doses are aimed to be <4mg per day), specifically when immunotherapies are utilized.

Ultimately, while it is important that surgeons be made cognizant of these reported adverse events, the current literature is not sufficiently clear as to whether this heightened risk of bleeding is significant enough to merit changes in perioperative management, or whether the risk of bleeding varies by primary tumor type. Although a rare event, the risk of intracranial bleeding associated with these newer agents is a serious and possibly fatal occurrence that requires further investigation to identify high‐risk patients that should not receive treatment. There is also a need to better define the risk of intracranial bleeding in patients with previously treated vs untreated BM.^195^ Further clinical trials with larger sample size are necessary to better identify the safety profile for patients with BM treated with various chemotherapeutic agents.

## 6 PRACTICES REGARDING SURGICAL MANAGEMENT OF BRAIN METASTASES AT THE AUTHORS' HOME INSTITUTION

- Based on the available literature, we offer resection for selected patients with 1‐2 target lesions (ie size > 3 cm and/or symptomatic) with well‐controlled primary disease (or high probability of responding to systemic therapy), good prognosis (generally greater than six months) and KPS > 70. Resection is carefully considered according to the following criteria in patients with multiple intracranial metastases: symptomatic patients without highly radiosensitive pathologies, RPA class 1 or 2, surgically accessible metastases amenable to gross total resection, and symptomatology less likely to be controlled with SRS (eg caused by lesion >3 cm, significant mass effect or edema resulting in >1 cm midline shift, brainstem compression, or significant hydrocephalus or threat thereof). Notably, this includes resection of BM that indirectly or even directly affect eloquent areas within the brain, as the goal in these instances is to utilize resection to rapidly restore function, though supporting data may be sparse.
- It is the opinion of the authors that adjuvant SRS should be considered as the first‐line option following resection (or as an alternative to resection when surgery is less appropriate), with WBRT reserved for patients who have multiple lesions with a cumulative tumor volume > 10 cm^3^, have nonlocalized disease such as SCLC or leptomeningeal disease, or as salvage therapy for those do not respond to SRS.
- We currently employ biopsy + LITT as a preferred early avenue for metastatic disease showing radiographic progression, whether RIDIR or recurrent disease.
- As routine practice in the peri‐operative and prophylactic setting, sequential compression devices are provided immediately postoperatively. Additionally, VTE chemoprophylactic anticoagulation with enoxaparin is initiated on postoperative day 1 or 2 (provider‐dependent, and generally dependent on tumor vascularity and difficulty with attaining intraoperative hemostasis) and continued until discharge or until return to prior ambulatory status. This is protocoled for patients without evidence of hemorrhage on postoperative imaging.
- Regarding therapeutic anticoagulation in BM patients with known VTE who undergo surgery, reinitiation of therapeutic dosing with enoxaparin (1mg/kg/twice daily) is generally implemented by postoperative day 7, to be continued until close follow‐up with the treating oncologist.
- Due to the risks associated with intracranial hemorrhage and impaired wound healing, bevacizumab (anti‐VEGF chemotherapeutic) is commonly held for 4‐6 weeks prior to proceeding with surgical interventions within the intracranial compartment. The half‐life of sunitinib or sorafenib is shorter (eg 40‐60 hours for sunitinib and 20‐48 hours for sorafenib), and these are typically held only 3‐4 days prior to surgery.

## 7 CONCLUSION

Surgery (whether biopsy, resection, or laser ablation) still maintains an important and evolving role in the management of BM. Studies continue to demonstrate that resection in appropriately selected patients results in improved functional and survival outcomes, particularly when combined with radiotherapies. Maximal, safe, *en bloc* resection, with additional consideration given to clinically and radiographically undetectable infiltrative cells, should be the goal of surgery in order to optimize outcome. The typical upfront treatment decision for newly diagnosed BM should be between SRS vs resection followed by postoperative SRS. Minimally invasive procedures, such as LITT, may supplant open surgery when lesions are otherwise surgically inaccessible, or represent recurrence/progression vs RIDIR. WBRT may supplant SRS when combined tumor volumes are large (>10 cc) or pathologies are nonfocal (ie SCLC). Adjuvant SRS or WBRT is absolutely necessary to reduce the risk of local and distant intracranial tumor recurrence after surgery. While routine, prophylactic AED are not recommended, perioperative use of dexamethasone and early initiation of mechanical and chemoprophylaxis against VTE is advised. Well‐designed, randomized controlled trials comparing the efficacy of SRS alone vs current surgical techniques with postoperative SRS are needed in this unique and increasingly prevalent patient population.

## ENDNOTES

1 Deep (e.g. medulla, pons, midbrain, thalamus, basal ganglia).

2 Eloquent (i.e. generally described as the primary motor, sensory, visual cortices and their associated fiber tracts, in addition to the deep structures previously mentioned).

## References

[1] Sperduto PW , Chao ST , Sneed PK , et al. Diagnosis‐specific prognostic factors, indexes, and treatment outcomes for patients with newly diagnosed brain metastases: a multi‐institutional analysis of 4259 patients. Int J Radiat Oncol Biol Phys. 2010;77(3):655‐661. 19942357 10.1016/j.ijrobp.2009.08.025

[2] Zimm S , Wampler GL , Stablein D , Hazra T , Young HF . Intracerebral metastases in solid‐tumor patients: natural history and results of treatment. Cancer. 1981;48(2):384‐394. 7237407 10.1002/1097-0142(19810715)48:2<384::aid-cncr2820480227>3.0.co;2-8

[3] Claus EB . Neurosurgical management of metastases in the central nervous system. Nat Rev Clin Oncol. 2012;9(2):79‐86. 10.1038/nrclinonc.2011.179 22143137

[4] Ostrom QT , Gittleman H , Fulop J , et al. Statistical report: primary brain and central nervous system tumors diagnosed in the United States in 2008–2012. Neuro Oncol. 2015;17(suppl 4):iv1‐iv62. 26511214 10.1093/neuonc/nov189 PMC4623240

[5] Colaco R , Martin P , Chiang V . Evolution of multidisciplinary brain metastasis management: case study and literature review. Yale J Biol Med. 2015;88(2):157‐165. 26029014 PMC4445437

[6] Steeg PS , Camphausen KA , Smith QR . Brain metastases as preventive and therapeutic targets. Nat Rev Cancer. 2011;11(5):352‐363. 21472002 10.1038/nrc3053 PMC7351203

[7] Shen CJ , Lim M , Kleinberg LR . Controversies in the therapy of brain metastases: shifting paradigms in an era of effective systemic therapy and longer‐term survivorship. Curr Treat Options Oncol. 2016;17(9):46. 27447703 10.1007/s11864-016-0423-3

[8] Schouten LJ , Rutten J , Huveneers HA , Twijnstra A . Incidence of brain metastases in a cohort of patients with carcinoma of the breast, colon, kidney, and lung and melanoma. Cancer. 2002;94(10):2698‐2705. 12173339 10.1002/cncr.10541

[9] Mut M . Surgical treatment of brain metastasis: a review. Clin Neurol Neurosurg. 2012;114(1):1‐8. 22047649 10.1016/j.clineuro.2011.10.013

[10] Barajas RF , Cha S . Metastasis in adult brain tumors. Neuroimaging Clin N Am. 2016;26(4):601‐620. 27712796 10.1016/j.nic.2016.06.008 PMC5104196

[11] Arbit E , Wroński M , Burt M , Galicich JH . The treatment of patients with recurrent brain metastases. A retrospective analysis of 109 patients with nonsmall cell lung cancer. Cancer. 1995;76(5):765‐773. 8625178 10.1002/1097-0142(19950901)76:5<765::aid-cncr2820760509>3.0.co;2-e

[12] Agazzi S , Pampallona S , Pica A , et al. The origin of brain metastases in patients with an undiagnosed primary tumour. Acta Neurochir (Wien). 2004;146(2):153‐157. 14963747 10.1007/s00701-003-0188-x

[13] Han HJ , Chang WS , Jung HH , Park YG , Kim HY , Chang JH . Optimal treatment decision for brain metastases of unknown primary origin: the role and timing of radiosurgery. Brain Tumor Res Treat. 2016;4(2):107‐110. 27867920 10.14791/btrt.2016.4.2.107 PMC5114180

[14] Nussbaum ES , Djalilian HR , Cho KH , Hall WA . Brain metastases: histology, multiplicity, surgery, and survival. Cancer. 1996;78(8):1781‐1788. 8859192

[15] Ranasinghe MG , Sheehan JM . Surgical management of brain metastases. Neurosurg Focus. 2007;22(3):E2. 10.3171/foc.2007.22.3.3 17608355

[16] Sperduto PW , Berkey B , Gaspar LE , Mehta M , Curran W . A new prognostic index and comparison to three other indices for patients with brain metastases: an analysis of 1960 patients in the RTOG database. Int J Radiat Oncol Biol Phys. 2008;70(2):510‐514. 17931798 10.1016/j.ijrobp.2007.06.074

[17] Moazami N , Rice TW , Rybicki LA , et al. Stage III non‐small cell lung cancer and metachronous brain metastases. J Thorac Cardiovasc Surg. 2002;124(1):113‐122. 12091816 10.1067/mtc.2002.121678

[18] Stark AM , Tscheslog H , Buhl R , Held‐Feindt J , Mehdorn HM . Surgical treatment for brain metastases: prognostic factors and survival in 177 patients. Neurosurg Rev. 2005;28(2):115‐119. 15609059 10.1007/s10143-004-0364-3

[19] Gaspar L , Scott C , Rotman M , et al. Recursive partitioning analysis (RPA) of prognostic factors in three Radiation Therapy Oncology Group (RTOG) brain metastases trials. Int J Radiat Oncol Biol Phys. 1997;37(4):745‐751. 9128946 10.1016/s0360-3016(96)00619-0

[20] Nieder C , Nestle U , Motaref B , Walter K , Niewald M , Schnabel K . Prognostic factors in brain metastases: should patients be selected for aggressive treatment according to recursive partitioning analysis (RPA) classes? Int J Radiat Oncol Biol Phys. 2000;46(2):297‐302. 10661335 10.1016/s0360-3016(99)00416-2

[21] Wolchok JD , Chiarion‐Sileni V , Gonzalez R , et al. Overall survival with combined Nivolumab and Ipilimumab in advanced melanoma. N Engl J Med. 2017;377(14):1345‐1356. 28889792 10.1056/NEJMoa1709684 PMC5706778

[22] Motzer RJ , Tannir NM , McDermott DF , et al. Nivolumab plus Ipilimumab versus Sunitinib in advanced renal‐cell carcinoma. N Engl J Med. 2018;378(14):1277‐1290. 29562145 10.1056/NEJMoa1712126 PMC5972549

[23] Ferrel E , Roehrig A , Kaya E , et al. Retrospective study of metastatic melanoma and renal cell carcinoma to the brain with multivariate analysis of prognostic pre‐treatment clinical factors. Int J Mol Sci. 2016;17(3):400. 26999120 10.3390/ijms17030400 PMC4813255

[24] Sampson JH , Carter JH , Friedman AH , Seigler HF . Demographics, prognosis, and therapy in 702 patients with brain metastases from malignant melanoma. J Neurosurg. 1998;88(1):11‐20. 9420067 10.3171/jns.1998.88.1.0011

[25] Rostami R , Mittal S , Rostami P , Tavassoli F , Jabbari B . Brain metastasis in breast cancer: a comprehensive literature review. J Neurooncol. 2016;127(3):407‐414. 26909695 10.1007/s11060-016-2075-3

[26] Sheehan JP , Sun MH , Kondziolka D , Flickinger J , Lunsford LD . Radiosurgery in patients with renal cell carcinoma metastasis to the brain: long‐term outcomes and prognostic factors influencing survival and local tumor control. J Neurosurg. 2003;98(2):342‐349. 12593621 10.3171/jns.2003.98.2.0342

[27] Wroński M , Arbit E , McCormick B , Wrónski M . Surgical treatment of 70 patients with brain metastases from breast carcinoma. Cancer. 1997;80(9):1746‐1754. 9351543 10.1002/(sici)1097-0142(19971101)80:9<1746::aid-cncr8>3.0.co;2-c

[28] Redmond AJ , DiLuna ML , Hebert R , et al. Gamma knife surgery for the treatment of melanoma metastases: the effect of intratumoral hemorrhage on survival. J Neurosurg. 2008;109(Suppl):99‐105. 19123895 10.3171/JNS/2008/109/12/S16

[29] Pollock BE , Brown PD , Foote RL , Stafford SL , Schomberg PJ . Properly selected patients with multiple brain metastases may benefit from aggressive treatment of their intracranial disease. J Neurooncol. 2003;61(1):73‐80. 12587798 10.1023/a:1021262218151

[30] Bernhardt D , Adeberg S , Bozorgmehr F , et al. Outcome and prognostic factors in single brain metastases from small‐cell lung cancer. Strahlenther Onkol. 2018;194(2):98‐106. 29085978 10.1007/s00066-017-1228-4

[31] Schild SE , Sio TT , Daniels TB , Chun SG , Rades D . Prophylactic cranial irradiation for extensive small‐cell lung cancer. J Oncol Pract. 2017;13(11):732‐738. 29125923 10.1200/JOP.2017.026765

[32] van Meerbeeck JP , Fennell DA , De Ruysscher DK . Small‐cell lung cancer. Lancet. 2011;378(9804):1741‐1755. 21565397 10.1016/S0140-6736(11)60165-7

[33] Nieder C , Andratschke N , Grosu AL , Molls M . Recursive partitioning analysis (RPA) class does not predict survival in patients with four or more brain metastases. Strahlenther Onkol. 2003;179(1):16‐20. 12540980 10.1007/s00066-003-1028-x

[34] Lagerwaard FJ , Levendag PC , Nowak PJ , Eijkenboom WM , Hanssens PE , Schmitz PI . Identification of prognostic factors in patients with brain metastases: a review of 1292 patients. Int J Radiat Oncol Biol Phys. 1999;43(4):795‐803. 10098435 10.1016/s0360-3016(98)00442-8

[35] Jakola AS , Gulati S , Nerland US , Solheim O . Surgical resection of brain metastases: the prognostic value of the graded prognostic assessment score. J Neurooncol. 2011;105(3):573‐581. 21660540 10.1007/s11060-011-0623-4 PMC3215882

[36] Wronski M , Lederman G . A randomized trial to assess the efficacy of surgery in addition to radiotherapy in patients with a single cerebral metastasis. Cancer. 1997;80(5):1002‐1004. 9307209 10.1002/(sici)1097-0142(19970901)80:5<1002::aid-cncr30>3.0.co;2-c

[37] Noordijk EM , Vecht CJ , Haaxma‐Reiche H , et al. The choice of treatment of single brain metastasis should be based on extracranial tumor activity and age. Int J Radiat Oncol Biol Phys. 1994;29(4):711‐717. 8040016 10.1016/0360-3016(94)90558-4

[38] Patchell RA , Tibbs PA , Walsh JW , et al. A randomized trial of surgery in the treatment of single metastases to the brain. N Engl J Med. 1990;322(8):494‐500. 2405271 10.1056/NEJM199002223220802

[39] Vecht CJ , Haaxma‐Reiche H , Noordijk EM , et al. Treatment of single brain metastasis: radiotherapy alone or combined with neurosurgery? Ann Neurol. 1993;33(6):583‐590. 8498838 10.1002/ana.410330605

[40] D'Andrea G , Palombi L , Minniti G , Pesce A , Marchetti P . Brain metastases: surgical treatment and overall survival. World Neurosurg. 2017;97:169‐177. 27667577 10.1016/j.wneu.2016.09.054

[41] Mintz AH , Kestle J , Rathbone MP , et al. A randomized trial to assess the efficacy of surgery in addition to radiotherapy in patients with a single cerebral metastasis. Cancer. 1996;78(7):1470‐1476. 8839553 10.1002/(sici)1097-0142(19961001)78:7<1470::aid-cncr14>3.0.co;2-x

[42] Metellus P , Bialecki E , Le Rhun E , Dhermain F . Neurosurgical and radiosurgical decision making in brain metastasis patients in the area of targeted therapies? Chin Clin Oncol. 2015;4(2):19. 26112805 10.3978/j.issn.2304-3865.2015.06.02

[43] Hart MG , Grant R , Walker M , Dickinson H . Surgical resection and whole brain radiation therapy versus whole brain radiation therapy alone for single brain metastases. Cochrane Database Syst Rev. 2005;1:Cd003292. 10.1002/14651858.CD003292.pub2 PMC6457740 15674905

[44] Kalkanis SN , Kondziolka D , Gaspar LE , et al. The role of surgical resection in the management of newly diagnosed brain metastases: a systematic review and evidence‐based clinical practice guideline. J Neurooncol. 2010;96(1):33‐43. 19960230 10.1007/s11060-009-0061-8 PMC2808516

[45] Halasz LM , Uno H , Hughes M , et al. Comparative effectiveness of stereotactic radiosurgery versus whole‐brain radiation therapy for patients with brain metastases from breast or non‐small cell lung cancer. Cancer. 2016;122(13):2091‐2100. 27088755 10.1002/cncr.30009 PMC4911286

[46] Sahgal A , Aoyama H , Kocher M , et al. Phase 3 trials of stereotactic radiosurgery with or without whole‐brain radiation therapy for 1 to 4 brain metastases: individual patient data meta‐analysis. Int J Radiat Oncol Biol Phys. 2015;91(4):710‐717. 25752382 10.1016/j.ijrobp.2014.10.024

[47] Patel KR , Prabhu RS , Kandula S , et al. Intracranial control and radiographic changes with adjuvant radiation therapy for resected brain metastases: whole brain radiotherapy versus stereotactic radiosurgery alone. J Neurooncol. 2014;120(3):657‐663. 25189789 10.1007/s11060-014-1601-4

[48] Xue J , Kubicek GJ , Grimm J , et al. Biological implications of whole‐brain radiotherapy versus stereotactic radiosurgery of multiple brain metastases. J Neurosurg. 2014;121(Suppl):60‐68. 25434938 10.3171/2014.7.GKS141229

[49] Churilla TM , Chowdhury IH , Handorf E , et al. Comparison of local control of brain metastases with stereotactic radiosurgery vs surgical resection: a secondary analysis of a randomized clinical trial. JAMA Oncol. 2019;5(2):243. 30419088 10.1001/jamaoncol.2018.4610 PMC6439566

[50] Kayama T , Sato S , Sakurada K , et al. Effects of surgery with salvage stereotactic radiosurgery versus surgery with whole‐brain radiation therapy in patients with one to four brain metastases (JCOG0504): a phase III, noninferiority, randomized controlled trial. J Clin Oncol. 2018;36(33):JCO2018786186. 10.1200/JCO.2018.78.6186 29924704

[51] Mahajan A , Ahmed S , McAleer MF , et al. Post‐operative stereotactic radiosurgery versus observation for completely resected brain metastases: a single‐centre, randomised, controlled, phase 3 trial. Lancet Oncol. 2017;18(8):1040‐1048. 28687375 10.1016/S1470-2045(17)30414-X PMC5560102

[52] Kępka L , Tyc‐Szczepaniak D , Bujko K , et al. Stereotactic radiotherapy of the tumor bed compared to whole brain radiotherapy after surgery of single brain metastasis: results from a randomized trial. Radiother Oncol. 2016;121(2):217‐224. 27793446 10.1016/j.radonc.2016.10.005

[53] Lamba N , Muskens IS , DiRisio AC , et al. Stereotactic radiosurgery versus whole‐brain radiotherapy after intracranial metastasis resection: a systematic review and meta‐analysis. Radiat Oncol. 2017;12(1):106. 28646895 10.1186/s13014-017-0840-x PMC5483276

[54] Hall MD , McGee JL , McGee MC , et al. Cost‐effectiveness of stereotactic radiosurgery with and without whole‐brain radiotherapy for the treatment of newly diagnosed brain metastases. J Neurosurg. 2014;121(Suppl):84‐90. 10.3171/2014.7.GKS14972 25434941

[55] Lester‐Coll NH , Dosoretz AP , Magnuson WJ , Laurans MS , Chiang VL , Yu JB . Cost‐effectiveness of stereotactic radiosurgery versus whole‐brain radiation therapy for up to 10 brain metastases. J Neurosurg. 2016;125(Suppl 1):18‐25. 27903191 10.3171/2016.7.GKS161499

[56] Yamamoto M , Kawabe T , Sato Y , et al. Stereotactic radiosurgery for patients with multiple brain metastases: a case‐matched study comparing treatment results for patients with 2–9 versus 10 or more tumors. J Neurosurg. 2014;121(Suppl):16‐25. 25434933 10.3171/2014.8.GKS141421

[57] Hirshman BR , Wilson BR , Ali MA , et al. Cumulative intracranial tumor volume augments the prognostic value of diagnosis‐specific graded prognostic assessment model for survival in patients with melanoma cerebral metastases. Neurosurgery. 2018;83(2):237‐244. 28973506 10.1093/neuros/nyx380

[58] Ali MA , Hirshman BR , Wilson B , et al. Survival patterns of 5750 stereotactic radiosurgery‐treated patients with brain metastasis as a function of the number of lesions. World Neurosurg. 2017;107(944–51):e1. 10.1016/j.wneu.2017.07.062 PMC5654648 28735121

[59] Hirshman BR , Wilson B , Ali MA , et al. Superior prognostic value of cumulative intracranial tumor volume relative to largest intracranial tumor volume stereotactic radiosurgery‐treated brain metastasis patients. Neurosurgery. 2018;82:473‐480. 28658940 10.1093/neuros/nyx225 PMC5745302

[60] Emery A , Trifiletti DM , Romano KD , Patel N , Smolkin ME , Sheehan JP . More than just the number of brain metastases: evaluating the impact of brain metastasis location and relative volume on overall survival after stereotactic radiosurgery. World Neurosurg. 2017;99:111‐117. 27919761 10.1016/j.wneu.2016.11.119

[61] Bowden G , Kano H , Caparosa E , et al. Gamma knife radiosurgery for the management of cerebral metastases from non‐small cell lung cancer. J Neurosurg. 2015;122(4):766‐772. 25658792 10.3171/2014.12.JNS141111

[62] Kim IK , Starke RM , McRae DA , et al. Cumulative volumetric analysis as a key criterion for the treatment of brain metastases. J Clin Neurosci. 2017;39:142‐146. 28089195 10.1016/j.jocn.2016.12.006

[63] Muacevic A , Wowra B , Siefert A , Tonn JC , Steiger HJ , Kreth FW . Microsurgery plus whole brain irradiation versus Gamma Knife surgery alone for treatment of single metastases to the brain: a randomized controlled multicentre phase III trial. J Neurooncol. 2008;87(3):299‐307. 18157648 10.1007/s11060-007-9510-4

[64] Roos DE , Smith JG , Stephens SW . Radiosurgery versus surgery, both with adjuvant whole brain radiotherapy, for solitary brain metastases: a randomised controlled trial. Clin Oncol (R Coll Radiol). 2011;23(9):646‐651. 21592754 10.1016/j.clon.2011.04.009

[65] Muacevic A , Kreth FW , Horstmann GA , et al. Surgery and radiotherapy compared with gamma knife radiosurgery in the treatment of solitary cerebral metastases of small diameter. J Neurosurg. 1999;91(1):35‐43. 10389878 10.3171/jns.1999.91.1.0035

[66] Rades D , Bohlen G , Pluemer A , et al. Stereotactic radiosurgery alone versus resection plus whole‐brain radiotherapy for 1 or 2 brain metastases in recursive partitioning analysis class 1 and 2 patients. Cancer. 2007;109(12):2515‐2521. 17487853 10.1002/cncr.22729

[67] Bindal AK , Bindal RK , Hess KR , et al. Surgery versus radiosurgery in the treatment of brain metastasis. J Neurosurg. 1996;84(5):748‐754. 8622147 10.3171/jns.1996.84.5.0748

[68] Schöggl A , Kitz K , Reddy M , et al. Defining the role of stereotactic radiosurgery versus microsurgery in the treatment of single brain metastases. Acta Neurochir (Wien). 2000;142(6):621‐626. 10949435 10.1007/s007010070104

[69] O'Neill BP , Iturria NJ , Link MJ , Pollock BE , Ballman KV , O'Fallon JR . A comparison of surgical resection and stereotactic radiosurgery in the treatment of solitary brain metastases. Int J Radiat Oncol Biol Phys. 2003;55(5):1169‐1176. 12654423 10.1016/s0360-3016(02)04379-1

[70] Ikushima H , Tokuuye K , Sumi M , et al. Fractionated stereotactic radiotherapy of brain metastases from renal cell carcinoma. Int J Radiat Oncol Biol Phys. 2000;48(5):1389‐1393. 11121638 10.1016/s0360-3016(00)00804-x

[71] Shinoura N , Yamada R , Okamoto K , Nakamura O , Shitara N . Local recurrence of metastatic brain tumor after stereotactic radiosurgery or surgery plus radiation. J Neurooncol. 2002;60(1):71‐77. 12416548 10.1023/a:1020256721761

[72] Wang LG , Guo Y , Zhang X , et al. Brain metastasis: experience of the Xi‐Jing hospital. Stereotact Funct Neurosurg. 2002;78(2):70‐83. 12566833 10.1159/000068015

[73] Lamba N , Cagney DN , Brigell RH , et al. Neurosurgical resection and stereotactic radiation versus stereotactic radiation alone in patients with a single or solitary brain metastasis. World Neurosurg. 2019;122:e1557‐e1561. 30471438 10.1016/j.wneu.2018.11.100

[74] Paek SH , Audu PB , Sperling MR , Cho J , Andrews DW . Reevaluation of surgery for the treatment of brain metastases: review of 208 patients with single or multiple brain metastases treated at one institution with modern neurosurgical techniques. Neurosurgery. 2005;56(5):1021‐1034; discussion ‐34. 15854250

[75] Al‐Shamy G , Sawaya R . Management of brain metastases: the indispensable role of surgery. J Neurooncol. 2009;92(3):275‐282. 19357955 10.1007/s11060-009-9839-y

[76] Prabhu RS , Press RH , Patel KR , et al. Single‐Fraction Stereotactic Radiosurgery (SRS) alone versus surgical resection and SRS for large brain metastases: a multi‐institutional analysis. Int J Radiat Oncol Biol Phys. 2017;99(2):459‐467. 28871997 10.1016/j.ijrobp.2017.04.006

[77] Masucci GL . Hypofractionated radiation therapy for large brain metastases. Front Oncol. 2018;8:379. 30333955 10.3389/fonc.2018.00379 PMC6176274

[78] Swift PS , Phillips T , Martz K , et al. CT characteristics of patients with brain metastases treated in RTOG study 79–16. Int J Radiat Oncol Biol Phys. 1993;25(2):209‐214. 8420868 10.1016/0360-3016(93)90341-r

[79] Tawbi HA , Forsyth PA , Algazi A , et al. Combined nivolumab and ipilimumab in melanoma metastatic to the brain. N Engl J Med. 2018;379(8):722‐730. 30134131 10.1056/NEJMoa1805453 PMC8011001

[80] Davies MA , Saiag P , Robert C , et al. Dabrafenib plus trametinib in patients with BRAF. Lancet Oncol. 2017;18(7):863‐873. 28592387 10.1016/S1470-2045(17)30429-1 PMC5991615

[81] Bachelot T , Romieu G , Campone M , et al. Lapatinib plus capecitabine in patients with previously untreated brain metastases from HER2‐positive metastatic breast cancer (LANDSCAPE): a single‐group phase 2 study. Lancet Oncol. 2013;14(1):64‐71. 23122784 10.1016/S1470-2045(12)70432-1

[82] Agboola O , Benoit B , Cross P , et al. Prognostic factors derived from recursive partition analysis (RPA) of Radiation Therapy Oncology Group (RTOG) brain metastases trials applied to surgically resected and irradiated brain metastatic cases. Int J Radiat Oncol Biol Phys. 1998;42(1):155‐159. 9747833 10.1016/s0360-3016(98)00198-9

[83] Bindal RK , Sawaya R , Leavens ME , Lee JJ . Surgical treatment of multiple brain metastases. J Neurosurg. 1993;79(2):210‐216. 8331402 10.3171/jns.1993.79.2.0210

[84] Buchsbaum JC , Suh JH , Lee SY , Chidel MA , Greskovich JF , Barnett GH . Survival by radiation therapy oncology group recursive partitioning analysis class and treatment modality in patients with brain metastases from malignant melanoma: a retrospective study. Cancer. 2002;94(8):2265‐2272. 12001126 10.1002/cncr.10426

[85] Iwadate Y , Namba H , Yamaura A . Significance of surgical resection for the treatment of multiple brain metastases. Anticancer Res. 2000;20(1B):573‐577. 10769728

[86] Kondziolka D , Lunsford LD , Flickinger JC . Controversies in the management of multiple brain metastases: the roles of radiosurgery and radiation therapy. Forum (Genova). 2001;11(1):47‐58. 11734864

[87] Kanner AA , Bokstein F , Blumenthal DT , Ram Z . Surgical therapies in brain metastasis. Semin Oncol. 2007;34(3):197‐205. 17560981 10.1053/j.seminoncol.2007.03.011

[88] Kondziolka D , Patel A , Lunsford LD , Kassam A , Flickinger JC . Stereotactic radiosurgery plus whole brain radiotherapy versus radiotherapy alone for patients with multiple brain metastases. Int J Radiat Oncol Biol Phys. 1999;45(2):427‐434. 10487566 10.1016/s0360-3016(99)00198-4

[89] Gaspar LE , Mehta MP , Patchell RA , et al. The role of whole brain radiation therapy in the management of newly diagnosed brain metastases: a systematic review and evidence‐based clinical practice guideline. J Neurooncol. 2010;96(1):17‐32. 19960231 10.1007/s11060-009-0060-9 PMC2808517

[90] Kocher M , Soffietti R , Abacioglu U , et al. Adjuvant whole‐brain radiotherapy versus observation after radiosurgery or surgical resection of one to three cerebral metastases: results of the EORTC 22952–26001 study. J Clin Oncol. 2011;29(2):134‐141. 21041710 10.1200/JCO.2010.30.1655 PMC3058272

[91] Patchell RA , Tibbs PA , Regine WF , et al. Postoperative radiotherapy in the treatment of single metastases to the brain: a randomized trial. JAMA. 1998;280(17):1485‐1489. 9809728 10.1001/jama.280.17.1485

[92] Ojerholm E , Lee J , Thawani JP , et al. Stereotactic radiosurgery to the resection bed for intracranial metastases and risk of leptomeningeal carcinomatosis. J Neurosurg. 2014;121(Suppl):75‐83. 10.3171/2014.6.GKS14708 25434940

[93] Wu SG , Sun JY , Tong Q , Li FY , He ZY . Clinical features of brain metastases in breast cancer: an implication for hippocampal‐sparing whole‐brain radiation therapy. Ther Clin Risk Manag. 2016;12:1849‐1853. 28008263 10.2147/TCRM.S124212 PMC5167295

[94] Harth S , Abo‐Madyan Y , Zheng L , et al. Estimation of intracranial failure risk following hippocampal‐sparing whole brain radiotherapy. Radiother Oncol. 2013;109(1):152‐158. 24100152 10.1016/j.radonc.2013.09.009

[95] Brown PD , Pugh S , Laack NN , et al. Memantine for the prevention of cognitive dysfunction in patients receiving whole‐brain radiotherapy: a randomized, double‐blind, placebo‐controlled trial. Neuro Oncol. 2013;15(10):1429‐1437. 23956241 10.1093/neuonc/not114 PMC3779047

[96] Hartford AC , Paravati AJ , Spire WJ , et al. Postoperative stereotactic radiosurgery without whole‐brain radiation therapy for brain metastases: potential role of preoperative tumor size. Int J Radiat Oncol Biol Phys. 2013;85(3):650‐655. 22795806 10.1016/j.ijrobp.2012.05.027

[97] Smith TR , Lall RR , Lall RR , et al. Survival after surgery and stereotactic radiosurgery for patients with multiple intracranial metastases: results of a single‐center retrospective study. J Neurosurg. 2014;121(4):839‐845. 24857242 10.3171/2014.4.JNS13789

[98] Luther N , Kondziolka D , Kano H , et al. Predicting tumor control after resection bed radiosurgery of brain metastases. Neurosurgery. 2013;73(6):1001‐1006; discussion 6. 24264235 10.1227/NEU.0000000000000148

[99] Brown PD , Ballman KV , Cerhan JH , et al. Postoperative stereotactic radiosurgery compared with whole brain radiotherapy for resected metastatic brain disease (NCCTG N107C/CEC·3): a multicentre, randomised, controlled, phase 3 trial. Lancet Oncol. 2017;18(8):1049‐1060. 28687377 10.1016/S1470-2045(17)30441-2 PMC5568757

[100] Atalar B , Modlin LA , Choi C , et al. Risk of leptomeningeal disease in patients treated with stereotactic radiosurgery targeting the postoperative resection cavity for brain metastases. Int J Radiat Oncol Biol Phys. 2013;87(4):713‐718. 24054875 10.1016/j.ijrobp.2013.07.034

[101] Cagney DN , Lamba N , Sinha S , et al. Association of neurosurgical resection with development of pachymeningeal seeding in patients with brain metastases. JAMA oncology. 2019;5(5):703. 30844036 10.1001/jamaoncol.2018.7204 PMC6512273

[102] Asher AL , Burri SH , Wiggins WF , et al. A new treatment paradigm: neoadjuvant radiosurgery before surgical resection of brain metastases with analysis of local tumor recurrence. Int J Radiat Oncol Biol Phys. 2014;88(4):899‐906. 24606851 10.1016/j.ijrobp.2013.12.013

[103] Patel KR , Burri SH , Asher AL , et al. Comparing preoperative with postoperative stereotactic radiosurgery for resectable brain metastases: a multi‐institutional analysis. Neurosurgery. 2016;79(2):279‐285. 26528673 10.1227/NEU.0000000000001096

[104] Prabhu RS , Miller KR , Asher AL , et al. Preoperative stereotactic radiosurgery before planned resection of brain metastases: updated analysis of efficacy and toxicity of a novel treatment paradigm. J Neurosurg. 2018;1‐8. 10.3171/2018.7.JNS181293 30554174

[105] Kamath SD , Kumthekar PU . Immune checkpoint inhibitors for the treatment of Central Nervous System (CNS) metastatic disease. Front Oncol. 2018;8:414. 30319977 10.3389/fonc.2018.00414 PMC6171475

[106] Amaral T , Tampouri I , Eigentler T , et al. Immunotherapy plus surgery/radiosurgery is associated with favorable survival in patients with melanoma brain metastasis. Immunotherapy. 2019;11(4):297‐309. 30606066 10.2217/imt-2018-0149

[107] Bindal RK , Sawaya R , Leavens ME , Hess KR , Taylor SH . Reoperation for recurrent metastatic brain tumors. J Neurosurg. 1995;83(4):600‐604. 7674007 10.3171/jns.1995.83.4.0600

[108] Vecil GG , Suki D , Maldaun MV , Lang FF , Sawaya R . Resection of brain metastases previously treated with stereotactic radiosurgery. J Neurosurg. 2005;102(2):209‐215. 15739546 10.3171/jns.2005.102.2.0209

[109] Al‐Zabin M , Ullrich WO , Brawanski A , Proescholdt MA . Recurrent brain metastases from lung cancer: the impact of reoperation. Acta Neurochir (Wien). 2010;152(11):1887‐1892. 20617447 10.1007/s00701-010-0721-7

[110] Zacest AC , Besser M , Stevens G , Thompson JF , McCarthy WH , Culjak G . Surgical management of cerebral metastases from melanoma: outcome in 147 patients treated at a single institution over two decades. J Neurosurg. 2002;96(3):552‐558. 11883841 10.3171/jns.2002.96.3.0552

[111] Ammirati M , Cobbs CS , Linskey ME , et al. The role of retreatment in the management of recurrent/progressive brain metastases: a systematic review and evidence‐based clinical practice guideline. J Neurooncol. 2010;96(1):85‐96. 19957016 10.1007/s11060-009-0055-6 PMC2808530

[112] Overett TK , Shiu MH . Surgical treatment of distant metastatic melanoma. Indications and results. Cancer. 1985;56(5):1222‐1230. 4016710 10.1002/1097-0142(19850901)56:5<1222::aid-cncr2820560544>3.0.co;2-a

[113] Peña LA , Fuks Z , Kolesnick RN . Radiation‐induced apoptosis of endothelial cells in the murine central nervous system: protection by fibroblast growth factor and sphingomyelinase deficiency. Cancer Res. 2000;60(2):321‐327. 10667583

[114] Hopewell JW , van der Kogel AJ . Pathophysiological mechanisms leading to the development of late radiation‐induced damage to the central nervous system. Front Radiat Ther Oncol. 1999;33:265‐275. 10549496 10.1159/000061239

[115] Van Eldik LJ , Thompson WL , Ralay Ranaivo H , Behanna HA , Martin WD . Glia proinflammatory cytokine upregulation as a therapeutic target for neurodegenerative diseases: function‐based and target‐based discovery approaches. Int Rev Neurobiol. 2007;82:277‐296. 17678967 10.1016/S0074-7742(07)82015-0

[116] Yoshii Y . Pathological review of late cerebral radionecrosis. Brain Tumor Pathol. 2008;25(2):51‐58. 18987829 10.1007/s10014-008-0233-9

[117] Li YQ , Chen P , Haimovitz‐Friedman A , Reilly RM , Wong CS . Endothelial apoptosis initiates acute blood‐brain barrier disruption after ionizing radiation. Cancer Res. 2003;63(18):5950‐5956. 14522921

[118] Siu A , Wind JJ , Iorgulescu JB , Chan TA , Yamada Y , Sherman JH . Radiation necrosis following treatment of high grade glioma–a review of the literature and current understanding. Acta Neurochir (Wien). 2012;154(2):191‐201; discussion. 22130634 10.1007/s00701-011-1228-6

[119] Narloch JL , Farber SH , Sammons S , et al. Biopsy of enlarging lesions after stereotactic radiosurgery for brain metastases frequently reveals radiation necrosis. Neuro Oncol. 2017;19(10):1391‐1397. 28472527 10.1093/neuonc/nox090 PMC5596170

[120] Jeon YS , Koh YC , Song SW , Cho J , Lim SD . Palliative resection of metastatic brain tumors previously treated by stereotactic radiosurgery. Brain Tumor Res Treat. 2016;4(2):116‐123. 27867922 10.14791/btrt.2016.4.2.116 PMC5114182

[121] Lagman C , Chung LK , Pelargos PE , et al. Laser neurosurgery: A systematic analysis of magnetic resonance‐guided laser interstitial thermal therapies. J Clin Neurosci. 2017;36:20‐26. 27838155 10.1016/j.jocn.2016.10.019

[122] Fabiano AJ , Alberico RA . Laser‐interstitial thermal therapy for refractory cerebral edema from post‐radiosurgery metastasis. World Neurosurg. 2014;81(3–4):652.e1‐4. 24140996 10.1016/j.wneu.2013.10.034

[123] Carpentier A , McNichols RJ , Stafford RJ , et al. Real‐time magnetic resonance‐guided laser thermal therapy for focal metastatic brain tumors. Oper Neurosurg. 2008;63:ONS21‐ONS29. 10.1227/01.neu.0000335007.07381.df 18728600

[124] Ali MA , Carroll KT , Rennert RC , et al. Stereotactic laser ablation as treatment for brain metastases that recur after stereotactic radiosurgery: a multiinstitutional experience. Neurosurg Focus. 2016;41(4):E11. 10.3171/2016.7.FOCUS16227 27690654

[125] Andrews DW , Scott CB , Sperduto PW , et al. Whole brain radiation therapy with or without stereotactic radiosurgery boost for patients with one to three brain metastases: phase III results of the RTOG 9508 randomised trial. Lancet. 2004;363(9422):1665‐1672. 15158627 10.1016/S0140-6736(04)16250-8

[126] Kohutek ZA , Yamada Y , Chan TA , et al. Long‐term risk of radionecrosis and imaging changes after stereotactic radiosurgery for brain metastases. J Neurooncol. 2015;125(1):149‐156. 26307446 10.1007/s11060-015-1881-3 PMC4726630

[127] Kumar AJ , Leeds NE , Fuller GN , et al. Malignant gliomas: MR imaging spectrum of radiation therapy‐ and chemotherapy‐induced necrosis of the brain after treatment. Radiology. 2000;217(2):377‐384. 11058631 10.1148/radiology.217.2.r00nv36377

[128] Murovic JA , Chang SD . The pathophysiology of cerebral radiation necrosis and the role of laser interstitial thermal therapy. World Neurosurg. 2015;83(1):23‐26. 24631912 10.1016/j.wneu.2014.03.015

[129] Sneed PK , Mendez J , Vemer‐van den Hoek J , et al. Adverse radiation effect after stereotactic radiosurgery for brain metastases: incidence, time course, and risk factors. J Neurosurg. 2015;123(2):373‐386. 25978710 10.3171/2014.10.JNS141610

[130] Shaw E , Scott C , Souhami L , et al. Single dose radiosurgical treatment of recurrent previously irradiated primary brain tumors and brain metastases: final report of RTOG protocol 90–05. Int J Radiat Oncol Biol Phys. 2000;47(2):291‐298. 10802351 10.1016/s0360-3016(99)00507-6

[131] Minniti G , Clarke E , Lanzetta G , et al. Stereotactic radiosurgery for brain metastases: analysis of outcome and risk of brain radionecrosis. Radiat Oncol. 2011;6:48. 21575163 10.1186/1748-717X-6-48 PMC3108308

[132] Korytko T , Radivoyevitch T , Colussi V , et al. 12 Gy gamma knife radiosurgical volume is a predictor for radiation necrosis in non‐AVM intracranial tumors. Int J Radiat Oncol Biol Phys. 2006;64(2):419‐424. 16226848 10.1016/j.ijrobp.2005.07.980

[133] Brandes AA , Tosoni A , Spagnolli F , et al. Disease progression or pseudoprogression after concomitant radiochemotherapy treatment: pitfalls in neurooncology. Neuro Oncol. 2008;10(3):361‐367. 18401015 10.1215/15228517-2008-008 PMC2563059

[134] Chamberlain MC , Glantz MJ , Chalmers L , Van Horn A , Sloan AE . Early necrosis following concurrent Temodar and radiotherapy in patients with glioblastoma. J Neurooncol. 2007;82(1):81‐83. 16944309 10.1007/s11060-006-9241-y

[135] de Wit MC , de Bruin HG , Eijkenboom W , Sillevis Smitt PA , van den Bent MJ . Immediate post‐radiotherapy changes in malignant glioma can mimic tumor progression. Neurology. 2004;63(3):535‐537. 15304589 10.1212/01.wnl.0000133398.11870.9a

[136] Chao ST , Ahluwalia MS , Barnett GH , et al. Challenges with the diagnosis and treatment of cerebral radiation necrosis. Int J Radiat Oncol Biol Phys. 2013;87(3):449‐457. 23790775 10.1016/j.ijrobp.2013.05.015

[137] Na A , Haghigi N , Drummond KJ . Cerebral radiation necrosis. Asia Pac J Clin Oncol. 2014;10(1):11‐21. 10.1111/ajco.12124 24175987

[138] Patel AJ , Suki D , Hatiboglu MA , et al. Factors influencing the risk of local recurrence after resection of a single brain metastasis. J Neurosurg. 2010;113(2):181‐189. 20035574 10.3171/2009.11.JNS09659

[139] Kamp MA , Slotty PJ , Cornelius JF , Steiger HJ , Rapp M , Sabel M . The impact of cerebral metastases growth pattern on neurosurgical treatment. Neurosurg Rev. 2018;41(1):77‐86. 27392678 10.1007/s10143-016-0760-5

[140] Tan TC , Black PM . Image‐guided craniotomy for cerebral metastases. Neurosurgery. 2007;61(suppl_1):82‐90. 12823876 10.1227/01.neu.0000068729.37362.f9

[141] Sawaya R , Hammoud M , Schoppa D , et al. Neurosurgical outcomes in a modern series of 400 craniotomies for treatment of parenchymal tumors. Neurosurgery. 1998;42(5):1044‐1055; discussion 55–6. 9588549 10.1097/00006123-199805000-00054

[142] Kondziolka D , Lunsford LD . Intraoperative navigation during resection of brain metastases. Neurosurg Clin N Am. 1996;7(2):267‐277. 8726440

[143] Yoshida S , Takahashi H . Cerebellar metastases in patients with cancer. Surg Neurol. 2009;71(2):184‐187. discussion 7. 18295837 10.1016/j.surneu.2007.10.010

[144] Suki D , Hatiboglu MA , Patel AJ , et al. Comparative risk of leptomeningeal dissemination of cancer after surgery or stereotactic radiosurgery for a single supratentorial solid tumor metastasis. Neurosurgery. 2009;64(4):664‐674. 19197219 10.1227/01.NEU.0000341535.53720.3E

[145] Baumert BG , Rutten I , Dehing‐Oberije C , et al. A pathology‐based substrate for target definition in radiosurgery of brain metastases. Int J Radiat Oncol Biol Phys. 2006;66(1):187‐194. 16814946 10.1016/j.ijrobp.2006.03.050

[146] Neves S , Mazal PR , Wanschitz J , et al. Pseudogliomatous growth pattern of anaplastic small cell carcinomas metastatic to the brain. Clin Neuropathol. 2001;20(1):38‐42. 11220694

[147] Yoo H , Kim YZ , Nam BH , et al. Reduced local recurrence of a single brain metastasis through microscopic total resection. J Neurosurg. 2009;110(4):730‐736. 19072310 10.3171/2008.8.JNS08448

[148] Volkov AA , Filis AK , Vrionis FD . Surgical treatment for leptomeningeal disease. Cancer Control. 2017;24(1):47‐53. 28178712 10.1177/107327481702400107

[149] Suki D , Abouassi H , Patel AJ , Sawaya R , Weinberg JS , Groves MD . Comparative risk of leptomeningeal disease after resection or stereotactic radiosurgery for solid tumor metastasis to the posterior fossa. J Neurosurg. 2008;108(2):248‐257. 18240919 10.3171/JNS/2008/108/2/0248

[150] Singh G , Rees JH , Sander JW . Seizures and epilepsy in oncological practice: causes, course, mechanisms and treatment. J Neurol Neurosurg Psychiatry. 2007;78(4):342‐349. 17369589 10.1136/jnnp.2006.106211 PMC2077803

[151] Mavrakis AN , Halpern EF , Barker FG , Gonzalez RG , Henson JW . Diagnostic evaluation of patients with a brain mass as the presenting manifestation of cancer. Neurology. 2005;65(6):908‐911. 16186533 10.1212/01.wnl.0000176059.21455.76

[152] Parker GM , Dunn IF , Ramkissoon SH , Eneman JD , Rabin MS , Arvold ND . Recurrent radiation necrosis in the brain following stereotactic radiosurgery. Pract Radiat Oncol. 2015;5(3):e151‐e154. 25432541 10.1016/j.prro.2014.10.006

[153] Forsyth PA , Weaver S , Fulton D , et al. Prophylactic anticonvulsants in patients with brain tumour. Can J Neurol Sci. 2003;30(2):106‐112. 12774949 10.1017/s0317167100053361

[154] Ansari SF , Bohnstedt BN , Perkins SM , Althouse SK , Miller JC . Efficacy of postoperative seizure prophylaxis in intra‐axial brain tumor resections. J Neurooncol. 2014;118(1):117‐122. 24532242 10.1007/s11060-014-1402-9 PMC4023013

[155] Wu AS , Trinh VT , Suki D , et al. A prospective randomized trial of perioperative seizure prophylaxis in patients with intraparenchymal brain tumors. J Neurosurg. 2013;118(4):873‐883. 23394340 10.3171/2012.12.JNS111970 PMC4083773

[156] Ryken TC , McDermott M , Robinson PD , et al. The role of steroids in the management of brain metastases: a systematic review and evidence‐based clinical practice guideline. J Neurooncol. 2010;96(1):103‐114. 19957014 10.1007/s11060-009-0057-4 PMC2808527

[157] Mikkelsen T , Paleologos NA , Robinson PD , et al. The role of prophylactic anticonvulsants in the management of brain metastases: a systematic review and evidence‐based clinical practice guideline. J Neurooncol. 2010;96(1):97‐102. 19957015 10.1007/s11060-009-0056-5 PMC2808526

[158] Alpert JS , Dalen JE . Epidemiology and natural history of venous thromboembolism. Prog Cardiovasc Dis. 1994;36(6):417‐422. 8184095 10.1016/s0033-0620(94)80050-2

[159] Horlander KT , Mannino DM , Leeper KV . Pulmonary embolism mortality in the United States, 1979–1998: an analysis using multiple‐cause mortality data. Arch Intern Med. 2003;163(14):1711‐1717. 12885687 10.1001/archinte.163.14.1711

[160] Goldhaber SZ , Visani L , De Rosa M . Acute pulmonary embolism: clinical outcomes in the International Cooperative Pulmonary Embolism Registry (ICOPER). Lancet. 1999;353(9162):1386‐1389. 10227218 10.1016/s0140-6736(98)07534-5

[161] Schiff D , DeAngelis LM . Therapy of venous thromboembolism in patients with brain metastases. Cancer. 1994;73(2):493‐498. 8293418 10.1002/1097-0142(19940115)73:2<493::aid-cncr2820730240>3.0.co;2-d

[162] Hutten BA , Prins MH , Gent M , Ginsberg J , Tijssen JG , Buller HR . Incidence of recurrent thromboembolic and bleeding complications among patients with venous thromboembolism in relation to both malignancy and achieved international normalized ratio: a retrospective analysis. J Clin Oncol. 2000;18(17):3078‐3083. 10963635 10.1200/JCO.2000.18.17.3078

[163] Palareti G , Legnani C , Lee A , et al. A comparison of the safety and efficacy of oral anticoagulation for the treatment of venous thromboembolic disease in patients with or without malignancy. Thromb Haemost. 2000;84(5):805‐810. 11127860

[164] Prandoni P , Lensing AW , Piccioli A , et al. Recurrent venous thromboembolism and bleeding complications during anticoagulant treatment in patients with cancer and venous thrombosis. Blood. 2002;100(10):3483‐3488. 10.1182/blood-2002-01-0108 12393647

[165] Koopman M , Prandoni P , Piovella F , et al. Treatment of venous thrombosis with intravenous unfractionated heparin administered in the hospital as compared with subcutaneous low‐molecular‐weight heparin administered at home. The Tasman Study Group. N Engl J Med. 1996;334(11):682‐687. 8594426 10.1056/NEJM199603143341102

[166] Levine M , Gent M , Hirsh J , et al. A comparison of low‐molecular‐weight heparin administered primarily at home with unfractionated heparin administered in the hospital for proximal deep‐vein thrombosis. N Engl J Med. 1996;334(11):677‐681. 8594425 10.1056/NEJM199603143341101

[167] Büller HR , Agnelli G , Hull RD , Hyers TM , Prins MH , Raskob GE . Antithrombotic therapy for venous thromboembolic disease. Chest. 2004;126(3):401S‐428S. 15383479 10.1378/chest.126.3_suppl.401S

[168] Lee A , Levine MN , Baker RI , et al. Low‐molecular‐weight heparin versus a coumarin for the prevention of recurrent venous thromboembolism in patients with cancer. N Engl J Med. 2003;349(2):146‐153. 12853587 10.1056/NEJMoa025313

[169] Raskob GE , van Es N , Verhamme P , et al. Edoxaban for the treatment of cancer‐associated venous thromboembolism. N Engl J Med. 2018;378(7):615‐624. 29231094 10.1056/NEJMoa1711948

[170] Donato J , Campigotto F , Uhlmann EJ , et al. Intracranial hemorrhage in patients with brain metastases treated with therapeutic enoxaparin: a matched cohort study. Blood. 2015;126(4):494‐499. 25987658 10.1182/blood-2015-02-626788 PMC4548746

[171] Weisberg LA . Hemorrhagic metastatic intracranial neoplasms: clinical‐computed tomographic correlations. Comput Radiol. 1985;9(2):105‐114. 3995932 10.1016/0730-4862(85)90006-x

[172] Kondziolka D , Bernstein M , Resch L , et al. Significance of hemorrhage into brain tumors: clinicopathological study. J Neurosurg. 1987;67(6):852‐857. 3316531 10.3171/jns.1987.67.6.0852

[173] Wakai S , Yamakawa K , Manaka S , Takakura K . Spontaneous intracranial hemorrhage caused by brain tumor: its incidence and clinical significance. Neurosurgery. 1982;10(4):437‐444. 7099393 10.1227/00006123-198204000-00004

[174] Morales A , Adelstein DJ . Should a patient with a brain tumor receive anticoagulation for a thromboembolic event? Cleve Clin J Med. 2001;68(1):13‐16. 11204362 10.3949/ccjm.68.1.13

[175] Alvarado G , Noor R , Bassett R , et al. Risk of intracranial hemorrhage with anticoagulation therapy in melanoma patients with brain metastases. Melanoma Res. 2012;22(4):310‐315. 22584956 10.1097/CMR.0b013e328353efd8 PMC4105847

[176] Dreyfus B , Kawabata H , Gomez A . Selected adverse events in cancer patients treated with vascular endothelial growth factor inhibitors. Cancer Epidemiol. 2013;37(2):191‐196. 23246035 10.1016/j.canep.2012.11.001

[177] Gordon CR , Rojavin Y , Patel M , et al. A review on bevacizumab and surgical wound healing: an important warning to all surgeons. Ann Plast Surg. 2009;62(6):707‐709. 19461291 10.1097/SAP.0b013e3181828141

[178] Kabbinavar F , Hurwitz HI , Fehrenbacher L , et al. Phase II, randomized trial comparing bevacizumab plus fluorouracil (FU)/leucovorin (LV) with FU/LV alone in patients with metastatic colorectal cancer. J Clin Oncol. 2003;21(1):60‐65. 12506171 10.1200/JCO.2003.10.066

[179] Saran F , Chinot OL , Henriksson R , et al. Bevacizumab, temozolomide, and radiotherapy for newly diagnosed glioblastoma: comprehensive safety results during and after first‐line therapy. Neuro Oncol. 2016;18(7):991‐1001. 26809751 10.1093/neuonc/nov300 PMC4896538

[180] Levin VA , Bidaut L , Hou P , et al. Randomized double‐blind placebo‐controlled trial of bevacizumab therapy for radiation necrosis of the central nervous system. Int J Radiat Oncol Biol Phys. 2011;79(5):1487‐1495. 20399573 10.1016/j.ijrobp.2009.12.061 PMC2908725

[181] Sandler A , Hirsh V , Reck M , von Pawel J , Akerley W , Johnson DH . An evidence‐based review of the incidence of CNS bleeding with anti‐VEGF therapy in non‐small cell lung cancer patients with brain metastases. Lung Cancer. 2012;78(1):1‐7. 22877947 10.1016/j.lungcan.2012.07.004

[182] Rogers LR . Cerebrovascular complications in patients with cancer. Semin Neurol. 2010;30(3):311‐319. 20577937 10.1055/s-0030-1255224

[183] Gordon MS , Margolin K , Talpaz M , et al. Phase I safety and pharmacokinetic study of recombinant human anti‐vascular endothelial growth factor in patients with advanced cancer. J Clin Oncol. 2001;19(3):843‐850. 11157038 10.1200/JCO.2001.19.3.843

[184] Carden CP , Larkin JM , Rosenthal MA . What is the risk of intracranial bleeding during anti‐VEGF therapy? Neuro Oncol. 2008;10(4):624‐630. 18539884 10.1215/15228517-2008-010 PMC2666237

[185] Oh Y , Wallace S , Taylor S , et al. Minimally increased risk of cerebrovascular occlusive disease or intracerebral hemorrhage in patients on bevacizumab treatment and association with intracerebral malignancies. J Clin Oncol. 2008;26(Suppl. 15 Pt I):628s.

[186] Besse B , Lasserre SF , Compton P , Huang J , Augustus S , Rohr UP . Bevacizumab safety in patients with central nervous system metastases. Clin Cancer Res. 2010;16(1):269‐278. 20028762 10.1158/1078-0432.CCR-09-2439

[187] Narita Y . Drug review: safety and efficacy of bevacizumab for glioblastoma and other brain tumors. Jpn J Clin Oncol. 2013;43(6):587‐595. 23585688 10.1093/jjco/hyt051

[188] Friedman HS , Prados MD , Wen PY , et al. Bevacizumab alone and in combination with irinotecan in recurrent glioblastoma. J Clin Oncol. 2009;27(28):4733‐4740. 19720927 10.1200/JCO.2008.19.8721

[189] Skillings JR , Johnson DH , Miller K , et al. Arterialthromboembolic events (ATEs) in a pooled analysis of 5 randomized, controlled trials (RCTs) of bevacizumab (BV) with chemotherapy. J Clin Oncol. 2005;23(16_suppl):3019.

[190] Scappaticci FA , Skillings JR , Holden SN , et al. Arterial thromboembolic events in patients with metastatic carcinoma treated with chemotherapy and bevacizumab. J Natl Cancer Inst. 2007;99(16):1232‐1239. 17686822 10.1093/jnci/djm086

[191] Clark AJ , Butowski NA , Chang SM , et al. Impact of bevacizumab chemotherapy on craniotomy wound healing. J Neurosurg. 2011;114(6):1609‐1616. 21142749 10.3171/2010.10.JNS101042

[192] Je Y , Schutz FA , Choueiri TK . Risk of bleeding with vascular endothelial growth factor receptor tyrosine‐kinase inhibitors sunitinib and sorafenib: a systematic review and meta‐analysis of clinical trials. Lancet Oncol. 2009;10(10):967‐974. 19767240 10.1016/S1470-2045(09)70222-0

[193] Pouessel D , Culine S . High frequency of intracerebral hemorrhage in metastatic renal carcinoma patients with brain metastases treated with tyrosine kinase inhibitors targeting the vascular endothelial growth factor receptor. Eur Urol. 2008;53(2):376‐381. 17825982 10.1016/j.eururo.2007.08.053

[194] Friedman CF , Proverbs‐Singh TA , Postow MA . Treatment of the immune‐related adverse effects of immune checkpoint inhibitors: a review. JAMA Oncol. 2016;2(10):1346‐1353. 27367787 10.1001/jamaoncol.2016.1051

[195] Letarte N , Bressler LR , Villano JL . Bevacizumab and central nervous system (CNS) hemorrhage. Cancer Chemother Pharmacol. 2013;71(6):1561‐1565. 23564377 10.1007/s00280-013-2155-4

